# Boosting Li^+^ Diffusion in Lithium-Rich Oxides through Intrinsic Structural Design: Insights and Design Principles

**DOI:** 10.1007/s40820-026-02099-7

**Published:** 2026-03-05

**Authors:** Lifeng Xu, Min Hong, Jingjing Guo, Fangming Shen, Da Xu, Jinjian Zhang, Ying Zhang, Jianhui Zheng, Jun Lu

**Affiliations:** 1 College of Chemical and Biological Engineering, Zhejiang University, Hangzhou, 310058 People’s Republic of China; 2 Quzhou Institute of Power Battery and Grid Energy Storage, Quzhou, 324100 People’s Republic of China; 3 Zhejiang Shidai Lithium Battery Materials Co., Ltd., Quzhou, 324100 People’s Republic of China; 4 Zhejiang Youngdream Li-ion Co., Ltd., Quzhou, 324100 People’s Republic of China; 5 Walker Department of Mechanical Engineering, The University of Texas at Austin, Austin, TX 78712 USA

**Keywords:** Li-rich oxides, Li^+^ diffusion, Structural modification, In situ characterization

## Abstract

Lithium-rich oxide cathodes present high specific capacities (> 250 mAh g^−1^) and wide operating voltage windows (2.0–4.8 V), making them promising candidates for next-generation high-energy batteries. Their practical deployment, however, is limited by sluggish ion transport kinetics that arise from inherent structural constraints, including confined two-dimensional diffusion channels, transition metal migration, and local lattice distortions. These structural perturbations narrow Li^+^ pathways, intensify cation mixing, and generate localized strain fields, collectively increasing the Li^+^ migration energy barrier. To facilitate the rational design of fast-kinetic lithium-rich oxides through intrinsic structural optimization, a comprehensive elucidation of the structure–diffusion interplay is presented, with emphasis on the roles of lattice distortion and oxygen redox chemistry in modulating Li^+^ pathways and associated energy barriers. Structural design strategies that aim to improve ionic diffusivity are systematically evaluated, including interface engineering, morphology-directed design, and the modulation of redox chemistry. Advanced operando characterization techniques that capture dynamic structural and chemical evolution are also described as essential tools for guiding precise structure–performance analysis. The mechanistic insights and integrated analytical approaches summarized in this review establish a robust conceptual foundation for engineering lithium-rich oxides with enhanced ion transport kinetics, thereby supporting the advancement of next-generation high-power battery technologies.

## Highlights

- Sluggish Li^+^ transport limits high-power output and fast charging in lithium-rich oxides, governed by intrinsic factors (crystal structure, distortion, and reaction kinetics) and external factors (cathode/electrolyte interface behavior, volumetric strain, and particle size distribution).
- Rate performance can be improved through interface engineering, targeted doping, particle morphology control, bulk structural optimization, and manipulation of redox chemistry to accelerate Li^+^ transport and stabilize electrochemical reactions.
- Understanding dynamic Li^+^ transport requires advanced operando characterization and multiscale computational modeling. Overcoming the capacity-kinetics paradox requires a mechanism-driven approach aimed at lowering the energy barriers for Li^+^ migration.

## Graphical abstract

**Figure:**
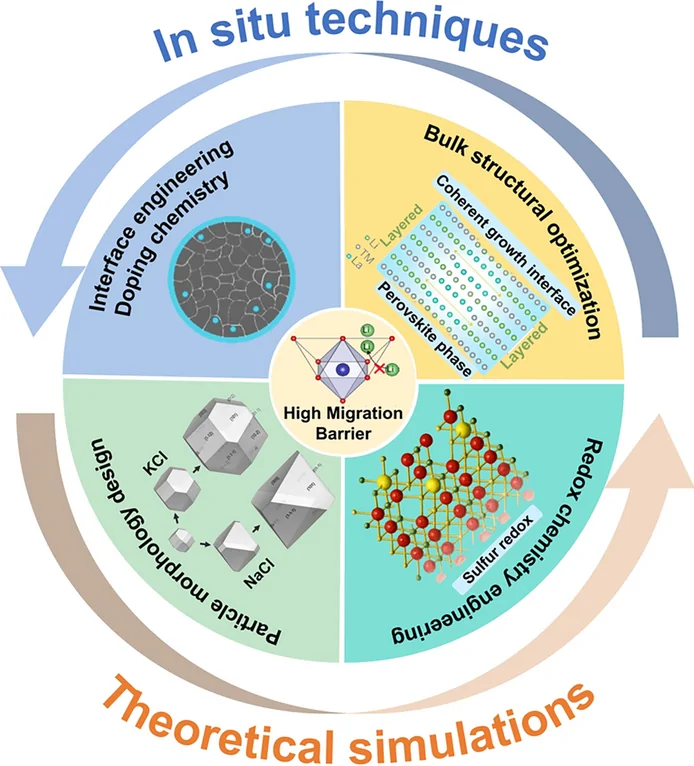

## Introduction

The rapid electrification of transportation and the growing integration of renewable energy into the power grid are driving a profound transformation of the global energy landscape. Lithium-ion batteries (LIBs) have become the dominant energy storage solution, powering the transition to sustainable energy due to their high energy efficiency, long cycle life, and scalable manufacturing. They are integral to a wide range of applications, from portable electronics to electric vehicles (EVs) and grid-scale storage [1]. However, the performance of state-of-the-art LIBs is inadequate for future demands, with energy densities typically below 400 Wh kg^−1^ and power density under 0.5 kW kg^−1^. This performance gap critically limits EV driving range, fast-charging capability, and grid stabilization services.

Overcoming the intrinsic limitations of conventional cathode materials, particularly layered transition metal oxides (LiMO_2_, M = Ni, Mn, Co), is a pressing priority [2]. These materials represent the primary bottleneck for improving capacity and voltage. Consequently, exploring new material systems that can simultaneously deliver ultra-high capacity, superior rate performance, and robust structural stability is imperative. Among the most promising candidates are lithium-rich oxides (LROs). Their unique ability to utilize both cationic (e.g., Ni^2+/4+^, Mn^3+/4+^, and Co^3+/4+^) redox and anionic (O^2−/n−^) redox chemistry enables reversible capacities exceeding 280 mAh g^−1^. This dual-redox mechanism offers a promising pathway to achieve energy densities surpassing those of layered Ni-rich oxides [3, 4]. However, these substantial capacity gains are counterbalanced by significant challenges, chief among which is the intrinsically sluggish kinetics of Li^+^ transport. This sluggish diffusion severely compromises fast-charging capability and energy efficiency [5].

The sluggish kinetic of LROs originates from a combination of inherent structural features and electrochemical derived structural degradation. During cycling, oxygen redox activity can trigger irreversible oxygen loss, transition metal (TM) migration into the Li layers, and phase transitions from layered to spinel or rocksalt structure. These degradation processes collapse low-barrier two-dimensional diffusion channels and generate tortuous, high-resistance migration pathways. In cation-disordered rocksalt (DRX) systems, the random cation distribution of Li and TM cations creates a heterogeneous energy landscape, raising the activation barrier for Li^+^ migration to over 0.5 eV [6–8]. Collectively, these phenomena depress the effective Li^+^ diffusivity to ~ 10^–15^–10^–14^ cm^2^ s^−1^, orders of magnitude lower than required for rapid energy delivery [9]. Microstructural defects further exacerbate kinetic limitations, leading to high overpotentials and efficiency loss. The problem is particularly acute in all-solid-state batteries, where sluggish bulk ion transport brings high interfacial impedance [10–12]. In the context of these multiscale kinetic constraints, “fast-kinetic lithium-rich oxides” refer to LROs capable of sustaining rapid Li^+^ transport under high-rate operation (> 2C-5C) while maintaining structural reversibility during deep delithiation. The intrinsic trade-off between achieving high capacities through combined anionic and cationic redox processes and preserving fast Li^+^ diffusion can be described as the “capacity-kinetics paradox,” in which the structural transformations that enable high capacity simultaneously impose severe kinetic restrictions. Recognition of this paradox underscores the importance of enhancing Li^+^ transport for the practical deployment of LROs-based energy storage systems.

**Fig. 1:**
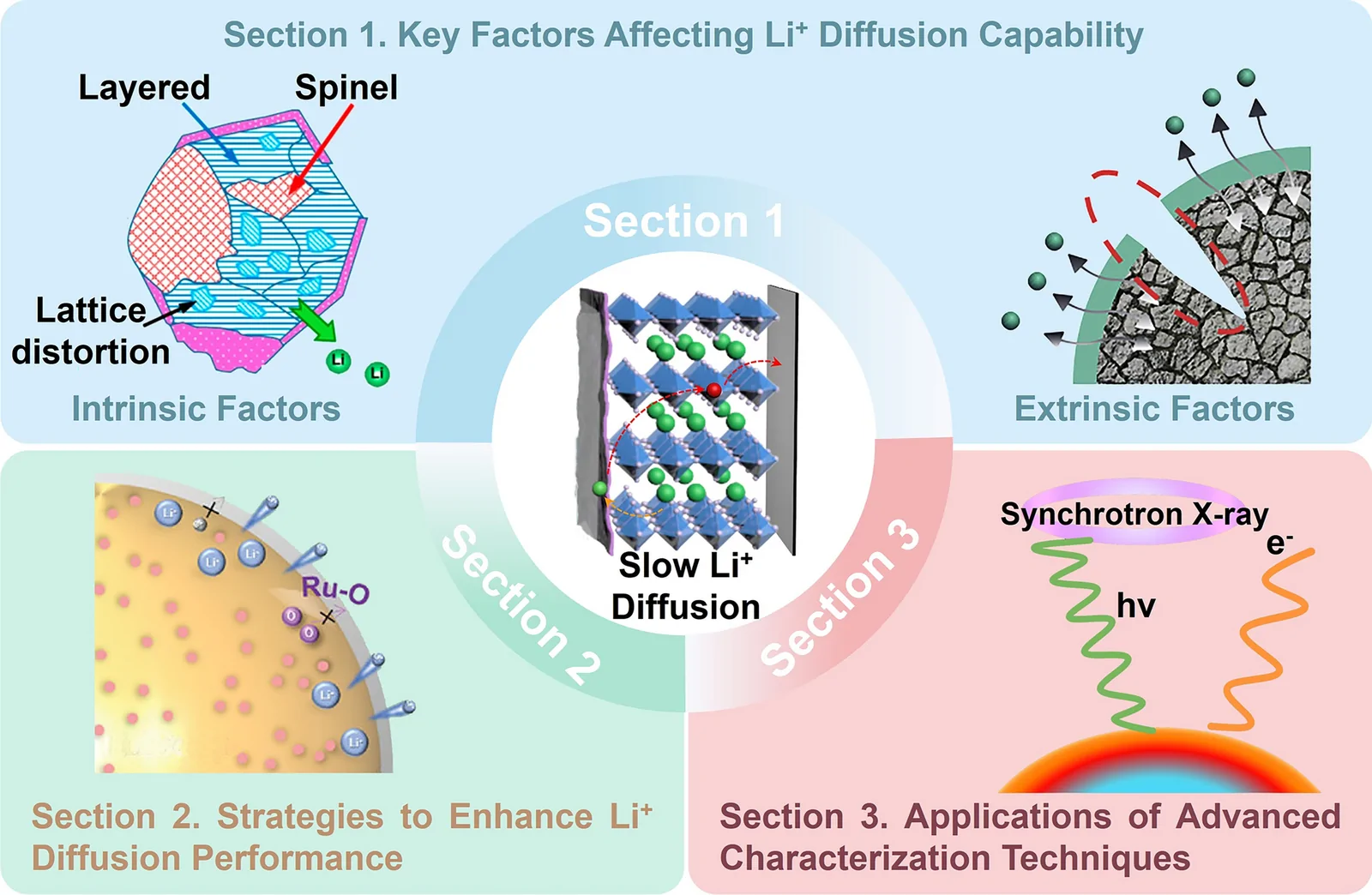
Overview of factors, strategies, and advanced characterization techniques related to Li^+^ diffusion in LROs

Given these challenges and motivated by the practical demand of industrial fast-charging targets (e.g., 60%–80% state of charge in 15–30 min) [13–15], this review provides a comprehensive overview of the fundamental ion transport limitations in LROs and highlights strategies to enhance their kinetics through intrinsic structural design, supported by modern analytical methodologies. Firstly, this review makes clear the essential structure–diffusion linkage in LROs by clarifying how specific structural features dictate Li^+^ transport behavior. Secondly, strategies for enhancing ionic conductivity and rate performance are thoroughly discussed and compared, encompassing interface engineering, morphological control, and redox regulation. In addition, modern characterization techniques that resolve dynamic structural and chemical evolution are demonstrated as powerful analytical methods for guiding structural design of LROs. Finally, actionable principles consolidated from the collective insights are provided to guide the structural design of high-rate, fast-charging LROs cathodes. As a roadmap for the discussion, Fig. 1 presents a conceptual framework outlining how intrinsic structural features in LROs restrict ion transport, along with corresponding structural-engineering strategies designed to enhance diffusion kinetics.

## Key Factors Affecting Li^+^ Diffusion Capability

**Fig. 2:**
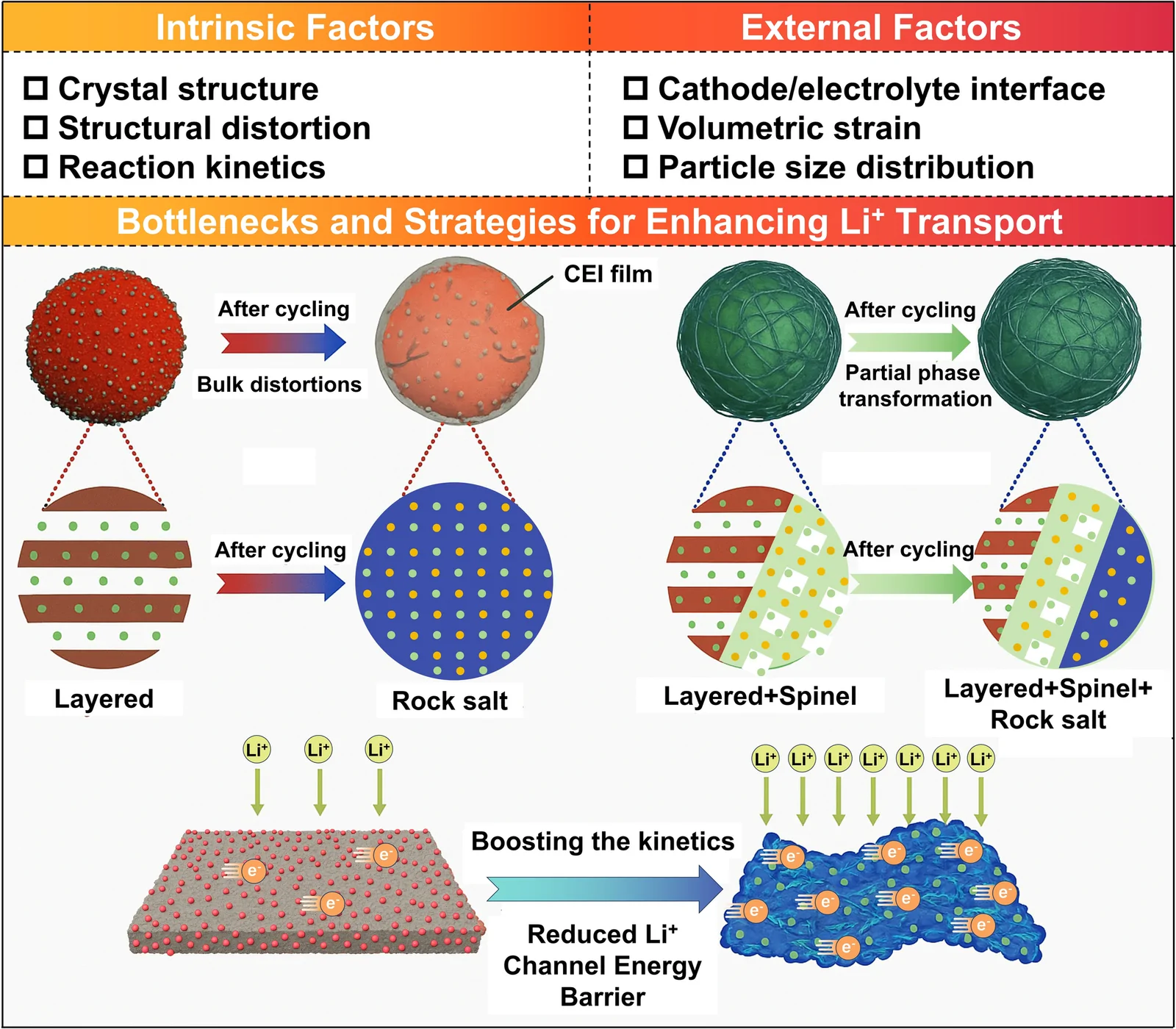
Bottlenecks and strategies for enhancing Li^+^ transport in LROs

The Li^+^ diffusion coefficient directly impacts the rate capabilities of LIBs, particularly in high-energy applications like EVs. This section examines both intrinsic and external factors that dictate Li^+^ diffusion in LROs, including atomic-level structural features, macroscopic properties, and electrochemical processes during the charge/discharge cycles, as summarized in Fig. 2.

### Intrinsic Factors

#### Crystal Structure

The crystal structure of LROs dictates the available Li^+^ diffusion pathways and strongly influences rate performance. From a transport perspective, cathode materials can be grouped into those that offer one-, two-, and three-dimensional diffusion channels. Olivine LiFePO_4_ allows Li^+^ transport exclusively along one-dimensional channels, requiring nanosized particles due to its low diffusion coefficient (~ 10^–16^–10^–15^ cm^2^ s^−1^). In contrast, layered LiMO_2_ (M = Co/Ni/Mn) and spinel LiMn_2_O_4_ provide two-dimensional and three-dimensional pathways, respectively, exhibiting significantly higher Li⁺ diffusivities (~ 10^–12^–10^–11^ and 10^–11^–10^–10^ cm^2^ s^−1^). Layered LROs fall in between: They possess two-dimensional Li layers similar to LiMO_2_ but suffer from much lower effective diffusion coefficients (~ 10^–15^–10^–14^ cm^2^ s^−1^) due to oxygen redox participation and pronounced structural evolution [16]. The Li^+^ diffusion characteristics in representative cathodes of different structural families are summarized in Table 1.

**Table 1:** Summary of Li^+^ diffusion characteristics in representative cathode materials with different structural families

Structural family	Migration channel type	$${D}_{{Li}^{+}} $$ (cm^2^ s^−1^)
Olivine LiFePO_4_	One-dimensional diffusion	~ 10^–16^–10^–15^
Layered LiMO_2_ (M = Co/Ni/Mn)	Two-dimensional diffusion	~ 10^–12^–10^–11^
Spinel LiMn_2_O_4_	Three-dimensional diffusion	~ 10^–11^–10^–10^
O3/O2-type	Vacancy-assisted TSH/IZH diffusion	~ 10^–15^–10^–14^
DRX	Percolating 0-TM octahedral-tetrahedral-octahedral hopping	~ 10^–16^–10^–15^
Li_2_MO_2_	Two-dimensional diffusion	–
Li_5_MO_4_	Three-dimensional diffusion	–

Typical O3-type layered LROs (xLi_2_MnO_3_·(1−x)LiMO_2_) have attracted considerable interest due to their structural similarity to conventional layered cathodes. They consist of two interpenetrating face-centered-cubic (FCC) sublattices formed by oxygen anions and Li/TM cations stacked along the cubic direction [17, 18]. These structures can be conceptualized as an intergrowth of LiMO_2_ (R $\overline{3 }$ m) and Li_2_MnO_3_ (C2/m), where the latter features a honeycomb-like superstructure with one-third of the TM sites occupied by Li^+^, providing additional Li reservoirs but also introducing cation ordering-induced bottlenecks in the Li layers [19–24]. O2-type LROs share similar in-plane cation arrangements but differ in their oxygen stacking (ABCB/ABAC) and are usually obtained through Li^+^/Na^+^ ion exchange from P2-type Na precursors. Compared with O3 phases, O2-type analogues better accommodate reversible TM migration and often display reduced voltage decay and improved structural stability during deep cycling [25–29].

**Fig. 3:**
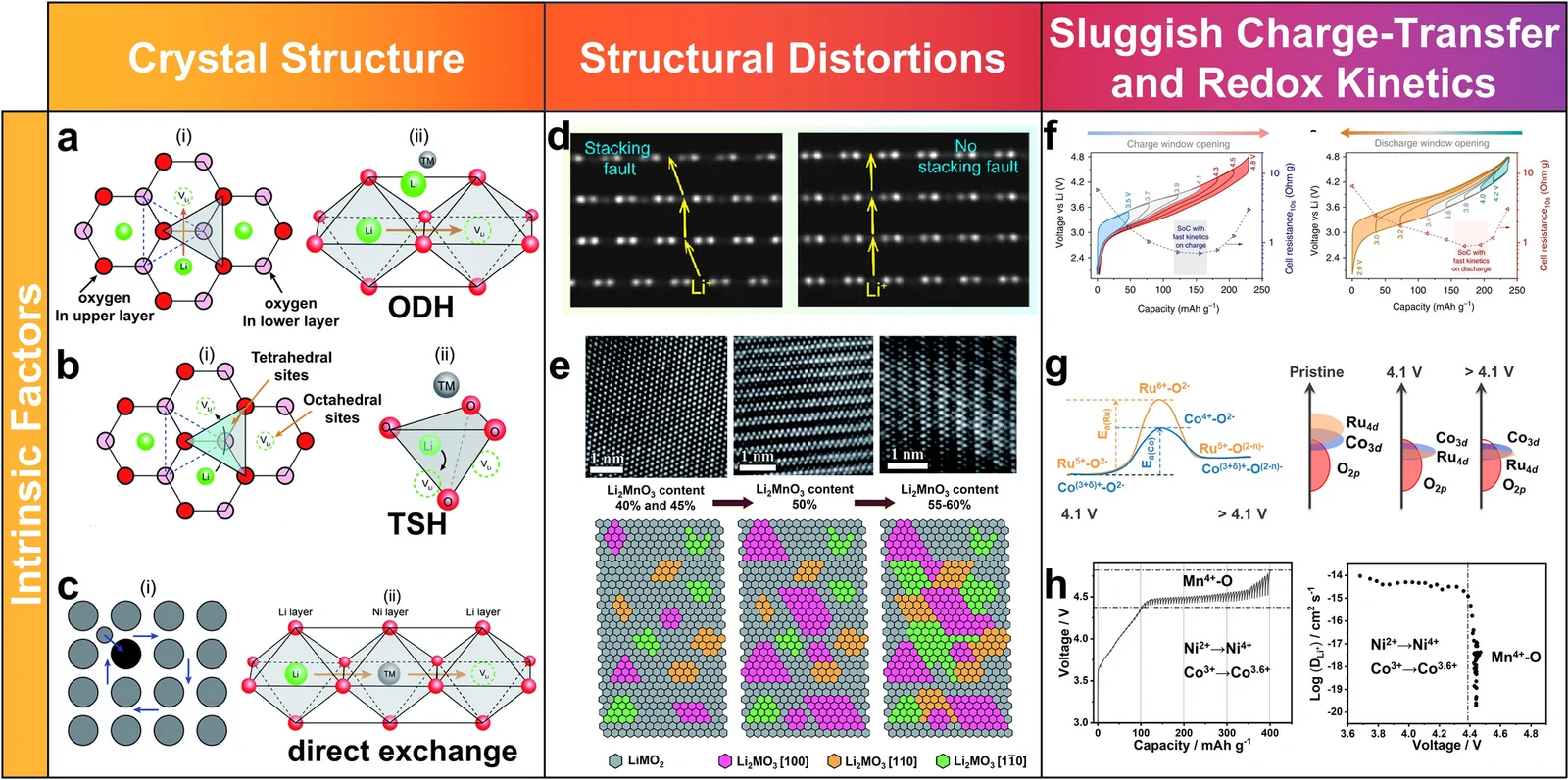
**a** ODH mechanism, where Li^+^ hops through the O–O dumbbell along the octahedral edge. **b** TSH mechanism, where Li^+^ moves from a tetrahedral site vacated by another Li^+^ and transitions to a neighboring octahedral site. **c** Direct exchange mechanism, where Li^+^ and TM ions exchange positions [30]. **d** Schematic illustrations of Li^+^ diffusion pathways in LROs with and without the stacking fault defect [33]. **e** Crystal structure evolution with increasing Li_2_MnO_3_ content [34]. **f** Hysteresis and path dependence in activated LROs studied by voltage window opening [35, 36]. **g** Anionic-cationic coupling in LROs [37]. **h** Slower kinetics of Mn-based reaction [36]

In both O3- and O2-type LROs, Li^+^ migration is primarily confined to the two-dimensional Li layers and proceeds via several competing mechanisms. At low vacancy concentrations, Li^+^ can hop directly between neighboring octahedral sites through the center of an oxygen–oxygen dumbbell (oxygen-dumbbell hopping, ODH, Fig. 3a) [30]. However, this straight path imposes a relatively high energy barrier because Li^+^ must squeeze through a narrow bottleneck defined by closely spaced oxygen ions. As vacancies accumulate during delithiation, Li^+^ preferentially follows a tetrahedral-assisted route (tetrahedral site hopping, TSH), in which it transiently occupies a face-sharing tetrahedral site before relaxing into a neighboring octahedron (Fig. 3b). This vacancy-assisted mechanism effectively combines interstitial and vacancy diffusion, generally lowering the migration barrier compared to pure ODH [30, 31]. More recently, a maximum-entropy reconstruction of in-operando neutron diffraction data has revealed that at high voltages, an indirect zigzag hopping (IZH) trajectory becomes dominant, providing a more efficient path by redistributing electrostatic repulsion, thus improving diffusion kinetics in heavily delithiated, structurally distorted lattices [32]. Beyond single-ion hopping, exchange-type mechanisms (Fig. 3c) involving concerted motion of multiple ions, such as Li/TM exchange via tetrahedral interstices and cooperative Li^+^ and Ni^2+^ migration, can form antisite defects and increase cation disorder [30]. These mechanisms raise Li^+^ migration barriers but also stabilize the structure and create new percolating channels for Li^+^ in disordered regions, presenting a trade-off between diffusion and structural integrity.

DRX materials represent another important structural family. They adopt an *α*-LiFeO_2_-type rocksalt framework (Fm $\overline{3 }$ m) in which Li and TM cations randomly occupy the octahedral sites of the FCC cation sublattice [6, 38]. This cation disorder generates a distribution of tetrahedral clusters (0-TM, 1-TM, 2-TM, 3-TM, 4-TM), and Li^+^ transport proceeds through an octahedral-tetrahedral-octahedral (o-t-o) hopping sequence. The most favorable, optimal migration channels are percolating 0-TM pathways, where the intermediate tetrahedron is coordinated only by Li. 1-TM tetrahedra are slightly less favorable, whereas 2-TM and higher-TM environments impose much larger barriers due to strong electrostatic repulsion from high-valence TM cations. Consequently, the macroscopic Li^+^ diffusivity in DRX materials is often limited to ~ 10^–16^–10^–15^ cm^2^ s^−1^, substantially lower than that of layered counterparts despite the presence of 3D percolation [7, 8]. The key advantage of DRX materials lies instead in their nearly isotropic structural response: volume changes during cycling are more homogeneous, mitigating layered-to-spinel transformations and collapse, thereby delivering superior long-term structural stability. The main disadvantage is sluggish Li^+^ transport unless a robust 0-TM percolation network is engineered by appropriate Li excess and TM selection.

LROs with higher Li/O ratios, such as Li_2_MO_2_ and Li_5_MO_4_, offer yet another design space in which diffusion dimensionality and migration barriers can be tuned [39, 40]. Orthorhombic Li_2_NiO_2_ (*Immm*), containing square-planar Ni coordination, provides predominantly 2D Li^+^ conduction along directions parallel to the b axis and diagonally within the a–b plane. However, this structure tends to undergo irreversible phase transitions during cycling, which undermines its practical rate performance despite potentially fast in-plane diffusion. In contrast, Li_5_FeO_4_ crystallizes in a defect-antifluorite (orthorhombic, *Pbca*) lattice with a 3D network of interconnected Li sites [41, 42]. Atomistic simulations suggest low activation energies (~ 0.3 eV) for Li^+^ migration along these 3D channels, indicating intrinsically favorable kinetics and defining these 3D paths as the optimal migration routes in this structure [43, 44]. The drawback is that such highly Li-rich frameworks often suffer from low intrinsic electronic conductivity and structural degradation at high states of charge. This point is mentioned because the reaction kinetics relies on coupled transport of Li^+^ and electrons; therefore, poor electronic conduction can increase polarization and hinder Li^+^ transport. Consequently, realizing their theoretical advantages requires careful control of particle morphology, sufficient electronic percolation, and an appropriate redox window.

Taken together, these different structural archetypes highlight that the optimal Li^+^ migration channel is highly structure-dependent: layered O3/O2 LROs benefit from vacancy-assisted TSH/IZH paths but are vulnerable to cation migration and oxygen redox-induced sluggish kinetics, DRX LROs rely on 0-TM o-t-o percolation networks that offer excellent structural robustness but limited diffusivity, and highly Li-rich antifluorite-like phases provide low-barrier 3D channels yet face challenges in electronic transport and phase stability.

#### Structural Distortions

Electrochemically induced structural distortions in LROs fundamentally alter Li^+^ diffusion pathways and elevate migration barriers, thereby limiting ionic transport and degrading electrochemical performance. This degradation is primary driven by the migration of TM ions into Li layers, a process that destabilizes the layered framework and generates stacking faults that block Li^+^ transport channels. These defects accumulate rapidly under high-voltage operation (> 4.5 V vs. Li^+^/Li), where the strong driving force for oxygen redox activity triggers lattice oxygen loss and promotes irreversible TM redistribution, as depicted in Fig. 3d [33, 45, 46]. Concomitantly, the formation of oxygen vacancies further destabilizes the anionic framework and exacerbates cation disorder, leading to additional lattice distortion and impaired Li^+^ mobility. Collectively, the synergistic coupling between TM migration and oxygen vacancy generation accelerates voltage decay and capacity fade, underscoring the kinetic and thermodynamic instability of LROs during extended cycling [47, 48].

Beyond these intrinsic instabilities, the composite nature of LROs introduces significant spatial heterogeneity. The coexistence of Li_2_MnO_3_-like and LiTMO_2_-like domains creates a heterogeneous atomic environment that impedes Li^+^ transport, as illustrated in Fig. 3e [34]. Specifically, the Li_2_MnO_3_-like regions, which are highly susceptible to Jahn–Teller distortions and cation disorder, exhibit higher Li^+^ migration barriers compared to the more ordered LiTMO_2_-like domains. This domain disparity induces fluctuations in bond lengths and local coordination environments, producing strain fields that obstruct Li^+^ transport at the atomic scale. During cycling, this atomic-scale strains manifest at the mesoscale as heterogeneous (de)lithiation and localized stress accumulation, ultimately leading to crack formation and particle fracture. Consequently, structural inhomogeneity of LROs not only limits ion transport efficiency but also couples it to mechanical degradation, thereby widening the gap between theoretical and practical performance [49, 50].

Electrostatic interactions and lattice distortions create significant barriers to Li^+^ transport, substantially limiting ionic mobility in LROs. Specifically, Li^+^ face strong repulsion at face-sharing sites with TM cations. This effect becomes particularly severe above 4.5 V, where oxygen lattice distorts and accumulated strain significantly amplify the repulsive forces. The formation of oxygen vacancies and tetrahedral distortions during anion redox further increases these repulsive forces, elevating the activation energy for Li^+^ migration [51, 52]. Concurrently, TM migration at particle surfaces promotes spinel-like reconstruction, progressively transforming the Li^+^ diffusion environment from a low-barrier 0-TM configuration to a high-barrier 3-TM configuration [53, 54]. Under fast charging, accumulated lattice strain severely retards Li^+^ kinetics and accelerates degradation [7, 46, 55]. These observations emphasize that both electronic and structural factors must be controlled to achieve stable Li^+^ transport [56].

To further refine this understanding, cross-scale evidence indicates that the kinetic limitations of LROs originate from the coupled evolution of local defect chemistry and mesoscale structural heterogeneity during anionic redox. At the local-structure level, anion-redox-induced distortion can stabilize Li occupation in tetrahedral environments, which interrupts the percolating diffusion network and increases the effective migration barrier under high-rate conditions. However, oxygen vacancy-related configurations can modify the Li migration landscape by destabilizing tetrahedral trapping and lowering the o-t-o hopping barrier, highlighting that defect formation is not only a degradation signature but also a kinetic lever when properly regulated [57]. At the particle scale, non-uniform anion activity and TM migration generate spatially heterogeneous strain and reconstruction, giving rise to distinct structural evolution pathways (e.g., progressive voiding/phase transformation during slow activation versus distortion-dominated lattice displacement under ultrafast (de)intercalation). These irreversible heterogeneities progressively increase polarization and impedance by disrupting continuous Li^+^ transport pathways [56]. Collectively, these results demonstrate that redox chemistry engineering must control not only the extent of anion participation but also the spatial distribution and reversibility of defect/strain evolution, in order to sustain rapid and stable Li^+^ transport.

In addition to layered LROs, O2-type LROs prepared by Na^+^/Li^+^ exchange face additional challenges. The high thermodynamic stability of P2-type structure and sluggish Na^+^/Li^+^ diffusion kinetics result in residual Na^+^ ions trapped in Li layers, which block Li^+^ transport channels [28]. While doping with Ni and Co can stabilize the layered structure and increase electronic conductivity, it may concurrently promote electrolyte decomposition, leading to thickened cathode-electrolyte interphase (CEI) and increased charge transfer resistance. Therefore, synergistic optimization of bulk doping and surface protection is critical to concurrently mitigate parasitic reactions and facilitate rapid Li⁺ transport [58, 59].

#### Sluggish Charge Transfer and Redox Kinetics

When LROs are charged to 4.5 V during initial activation, oxygen redox occurs in the Li_2_MnO_3_ phase, leading to oxygen vacancy formation and c-axis expansion. In theory, these structural changes should facilitate Li^+^ migration. However, a marked decline in the apparent Li^+^ diffusion coefficient ${D}_{{\mathrm{Li}}^{+}}$ is observed [60]. This sharp decrease in ${D}_{{\mathrm{Li}}^{+}}$ reveals that the primary limiting factor for Li^+^ transport has shifted from bulk ion migration to interfacial charge transfer process. As a result, the slow charge transfer process restricts the effective transport of Li^+^, impacting the overall performance of the battery.

Electrochemical performance of LROs at high states of charge is primarily governed by the kinetic interplay between cationic and anionic redox reactions. Compared to the conventional cation redox couples (Ni^2+^/Ni^4+^ and Co^3+^/Co^4+^), the oxygen redox process is inherently sluggish, particularly under high-voltage and high-current conditions (Fig. 3f, g**)**. This sluggish kinetics results in increased charge transfer resistance and severe polarization, which impedes Li^+^ diffusion and degrades the rate capability [35]. Impedance spectroscopy confirms a sharp increase in charge transfer resistance above 4.4 V, indicating a transition from ion diffusion limitations to anion-involved charge transfer processes. Further insights from Operando X-ray absorption spectroscopy combined with galvanostatic intermittent titration technique (GITT) reveal that the reaction kinetics of Mn are notably slower than those of Ni and Co (Fig. 3h) [36]. This kinetic hindrance is attributed to the structural disordering, Mn ion migration, and oxygen evolution during electrochemical activation. In conclusion, the slow kinetics of the anionic redox reaction, combined with the limitations associated with Mn, profoundly impact the overall electrochemical performance of LROs.

### Extrinsic Factors

#### Particle Size Distribution

**Fig. 4:**
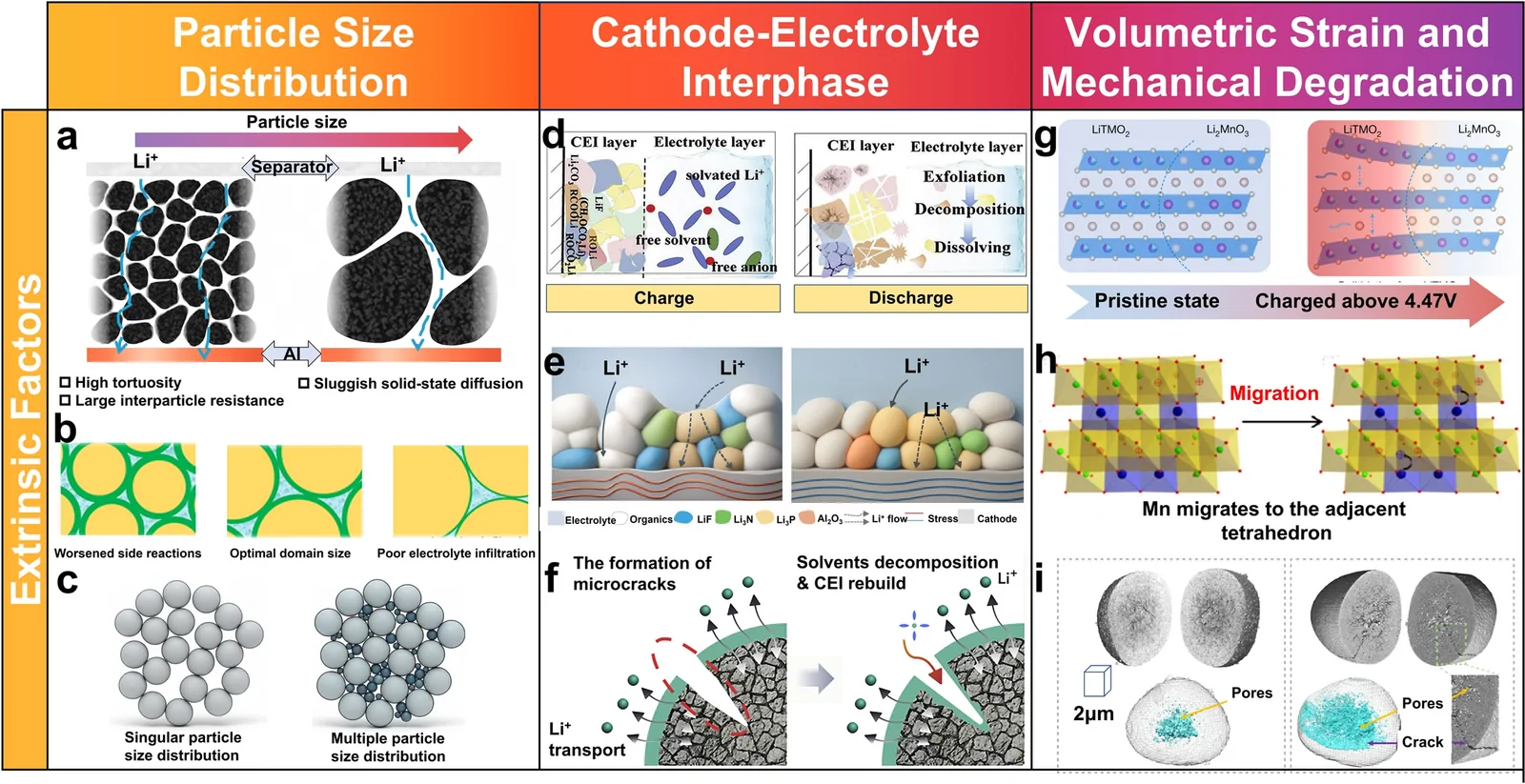
**a** Comparison of high tortuosity regular particle electrode and low-tortuosity chunky particle electrode [62]. **b** Schematic diagram of the domain size effects to the degraded mechanism [63]. **c** Particle size distribution [64]. **d, e** Dynamic evolution of the CEI during cycling and **f** schematic diagram of the reconstruction process of the CEI at the cathode crack [65]. **g** Strain-induced structural degradation [66]. **h** Phase evolution [67]. **i** Bulk crack formation [56]

Particle size distribution (PSD) is critical in determining the electrochemical performance of LROs, as it directly governs ion diffusion kinetics, structural stability of the electrode, and interfacial reactivity, all of which are key factors influencing capacity, rate capability, and cycling stability [61]. The PSD dictates the packing arrangement of active particles, thereby affecting ion diffusion pathways and transport efficiency within the electrode. In conventional electrodes composed of randomly stacked active particles, the disorderly arrangement of nanoparticles coupled with low packing density leads to high tortuosity and increased effective electrode thickness. As illustrated in Fig. 4a, this substantially prolongs the ion diffusion pathway and raises transport resistance. Studies show that tortuosity (*τ*), defined as the square of the ratio between the actual transport distance and the electrode thickness, is directly correlated with ion migration efficiency. Optimizing the PSD can reduce tortuosity, thereby facilitating ion diffusion and enhancing charge transfer kinetics. An ideal electrode structure should achieve dense packing of active materials while retaining adequate void space to minimize electrode thickness and accommodate volume variations during cycling.

PSD optimization also significantly influences electrode microstructure. Reducing particle size increases the specific surface area, offering more active sites for ion reactions and lowering solid-state diffusion resistance. However, as the average particle size increases or the PSD broadens, the overall energy density of the battery tends to decrease. Thus, selecting an appropriate PSD breadth and controlling particle size distribution are essential for enhancing electrode energy density. The electrochemical implications of PSD are dual-faceted: Smaller particles, owing to their higher surface area, promote faster (de)intercalation kinetics and improved rate performance. Yet, this increased surface area also intensifies side reactions with the electrolyte, accelerating CEI formation and structural degradation, particularly under high-voltage conditions (Fig. 4b) [63]. In contrast, larger particles exhibit lower surface reactivity, which enhances structural integrity and mitigates degradation. However, the longer diffusion paths inherent to larger particles result in sluggish ion transport, impairing rate performance [68].

Therefore, a balanced PSD combining small and large particles is essential for optimizing electrode performance [64]. To realize this in practice, an ideal architecture should incorporate a well-designed bimodal PSD. This can be achieved by intentionally blending large particles (e.g., D50: 10–20 μm) that act as a structural skeleton to ensure high tap density and long-term cycle stability, with a fraction of small particles (e.g., D50: 1–3 μm) that provide high specific surface area for rapid Li^+^ insertion and enhanced capacity. Such a design requires precise control during synthesis (e.g., via regulated nucleation/growth in co-precipitation) or post-treatment (e.g., controlled blending and deagglomeration). A well-controlled multimodal PSD improves particle packing, promotes efficient ion diffusion in the electrolyte, reduces electrode polarization, and facilitates Li^+^ intercalation kinetics, thus collectively improving rate capability and cycling stability. To validate this, performance must be correlated with quantitative metrics such as electrode density, porosity, and Li^+^ diffusion coefficients, alongside direct microstructural observation, as conceptually illustrated in Fig. 4c.

#### Cathode-Electrolyte Interphase

The CEI layer is a complex and dynamic interface that governs the ionic conductivity and electrochemical stability of LROs [69]. During the initial activation of LROs at voltages above 4.5 V, the decomposition of LiPF_6_-based organic carbonate electrolytes leads to the formation of a heterogeneous CEI layer, typically 6–10 nm thick, with non-uniform morphology, as shown in Fig. 4d, e [70, 71]. This non-uniform CEI layer gives rise to significant interfacial issues. A thick CEI layer prolongs the Li^+^ diffusion pathway and promotes interfacial polarization, and its heterogeneity also cause uneven electric field distributions, accelerating mechanical and chemical degradation of surface lattice. In contrast, a thin CEI layer, while facilitating rapid Li^+^ transport, often lacks mechanical integrity and chemical passivation capability. This makes it prone to fracture during cycling and trapping the interface in a continuous in a continuous cycle of side reactions and CEI reformation, thereby compromising interfacial stability, as shown in Fig. 4f [72–75]. Especially, at high voltages above 4.5 V, unique anion redox reactions produce reactive oxygen species (O^n−^), which react with carbonate solvents to form byproducts, further obstructing the reversible Li^+^ in/detercalation and exacerbating rate performance [76].

Constructing a CEI layer with moderate thickness (3–5 nm) and chemical/mechanical stability is essential to achieving interfacial stability and uniform Li^+^ diffusion [25]. Through electrolyte additives and interface engineering strategies, recent research has focused on pre-forming robust artificial CEI layers, particularly those enriched LiF. Due to its high mechanical modulus, wide electrochemical stability window, and low Li^+^ diffusion barrier, such LiF-enriched CEI layers significantly suppress impedance growth, maintain interfacial integrity over more than 1000 cycles, and enable capacity retention of > 90% [71, 77, 78].

#### Volumetric Strain and Mechanical Degradation

The performance degradation of LROs during electrochemical cycling is closely related to their multiscale structural evolution. During Li^+^ de/intercalation, the expansion along the c-axis is significantly greater than the contraction along a- and b-axes, leading to inhomogeneous stress distribution within the lattice, as depicted in Fig. 4g [66]. This crystallographic anisotropy induces substantial internal mechanical stresses that progressively erode the structural integrity, change local atomic environments, and perturb the optimal pathways for Li^+^ diffusion. Repeated lattice breathing, exacerbated under high-voltage operation where oxygen anions participate in redox, leads to irreversible oxygen loss. This creates vacancy clusters, destabilizes the lattice framework, and promotes migration of TM ions (e.g., Mn^4+^ and Ni^2+^) from TM layers into Li layers (Fig. 4h) [67, 79, 80]. This cation mixing induces disorder and initiates phase transitions from a layered to spinel or rocksalt structure. These atomic-scale rearrangements raise the energy barrier for Li^+^ migration, disrupt ionic percolation networks, and contribute to voltage fade. At the particle level, anisotropic strain manifests as accumulated internal stress and microcrack propagation (Fig. 4i). The stress concentration between primary particles during cycling can induce microcracking. For instance, in layered NCM materials, the c-axis expansion (from ~ 14.25 to ~ 14.40 Å) coupled with a/b-axis contraction results in volume changes of ~ 1.2% in NCM111 and up to ~ 5.1% in NCM811 [81, 82]. The localized stress fields generated during cycling can induce microcracks along grain boundaries. The synergy of structural heterogeneity, anisotropic volumetric strain, and microcrack propagation creates a highly heterogeneous mechanical and electrochemical environment across the electrode, posing a critical challenge to the rate capability and long-term cycling stability of LROs. These multiscale phenomena underscore that volumetric strain is not merely a mechanical by-product, but a pivotal factor linking atomic-scale lattice distortions with macroscopic performance degradation.

In summary, the degradation of LROs is governed by an interplay of mechanisms across different scales. Intrinsic structural distortions (lattice strain, cation mixing), the evolution of the CEI, and mechanically induced microcracks do not occur in isolation; they interact and amplify each other during cycling, collectively reshaping the energy landscape for Li^+^ migration. For instance, microcracks create fresh surfaces that accelerate CEI growth and parasitic reactions, while oxygen loss and phase transitions weaken the mechanical strength, facilitating further crack propagation. This intricate, multiscale coupling establishes a self-reinforcing degradation loop. Therefore, mitigating these detrimental effects requires a co-design philosophy that addresses mechanical robustness and efficient Li^+^ transport concurrently from the material’s inception. Preventing the formation of microcrack networks is paramount, as they not only fragment the electrode microstructure but also sever critical Li^+^ diffusion pathways, exacerbating polarization and capacity fade. This fundamental understanding directly informs the integrated material engineering strategies presented in Sect. 3, where approaches such as lattice doping, gradient architectures, and conformal surface coatings are employed in a coordinated manner to manage strain, stabilize interfaces, and maintain high ionic conductivity.

## Strategies to Enhance Li^+^ Diffusion Performance

To address the inherent limitations of LROs and achieve their practical viability, researchers have proposed multiple strategies for improving Li^+^ diffusion performance, primarily focusing on four key directions: interface engineering and doping chemistry, particle morphology design, bulk structural optimization and redox chemistry regulation. These strategies focus on enhancing ionic conductivity, stabilizing crystal structure, and mitigating interfacial side reactions.

### Interface Engineering and Doping Chemistry

**Fig. 5:**
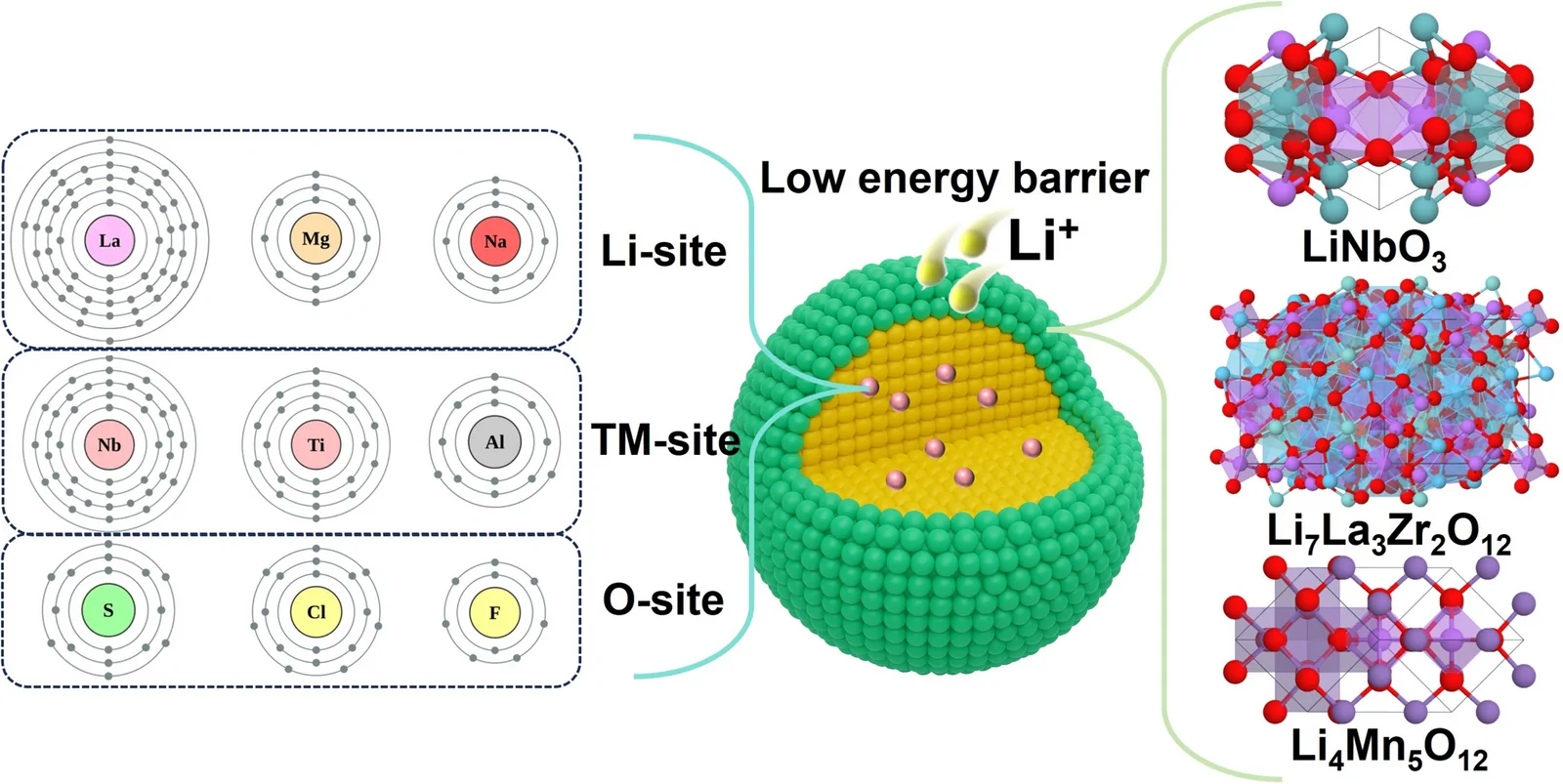
Interface engineering and doping chemistry for designing high-rate performance LROs

Interface engineering and doping chemistry have emerged as prevalent strategies for performance optimization in LROs, effectively addressing limitations in both surface and bulk properties (Fig. 5**)**. Through tailored surface coatings and selective bulk doping, these approaches synergistically enhance Li^+^ diffusion kinetics, reinforce structural integrity, and suppress interfacial side reactions-three fundamental requirements for advancing electrochemical performance of LROs. By employing this dual-modification approach, both ionic conductivity and high-rate capability are significantly improved while maintaining exceptional cycling stability, as summarized in Table 2.

**Table 2:** Rate performance of LROs modified by different interface and doping approaches

Modification methods	Cathode	Key mechanism	Voltage (V) and loading (mg cm^−2^)	Capacity (mAh g^−1^)	Cycling performance (mAh g^−1^)	Rate performance (mAh g^−1^)	${D}_{{\mathrm{Li}}^{+}}$ (cm^2^ s^−1^)
Surface Modification Only	La_0.8_Sr_0.2_MnO_3−y_ [83]	enhanced electronic conductivity	2.0–4.75 ~ 4.0	243.5, 0.1C (1C = 260 mA g^−1^)	202.0, 200 cycles, 1C (94.0%)	147.0, 5C	–
	5%LiNbO_3_ [84]	enhanced diffusion kinetics	2.0–4.8 ~ 2.8	279.8, 0.1C (1C = 250 mA g^−1^)	161.5, 250 cycles, 1C (74.0%)	143.4, 5C	4.121 × 10^–13^ after 25 cycles and 1.385 × 10^–13^ after 250 cycles
	spinel/layered Li_1.15_Ni_0.2_Mn_0.87_O_2_ hybrid nanofibers [85]	enhanced diffusion kinetics	2.0–4.8 ~ 1.2	300, 0.1C (1C = 250 mA g^−1^)	258.0, 70 cycles, 0.2C (> 80.0%)	150.0, 5C	8.03 × 10^−10^ at ≈ 4.5 V
	3%LiCeO_2_ [86]	enhanced diffusion kinetics	2.0–4.8 /	260.8, 0.1C (1C = 250 mA g^−1^)	176.3, 200 cycles, 1C (84.3%)	190.5, 5C 160.6, 10C	10^–14^
	Li_4_Mn_5_O_12_ and MgF_2_ [87]	enhanced diffusion kinetics	2.0–4.8 /	267.3, 0.1C (1 C = 200 mA g^−1^)	185.6, 300 cycles, 0.5C (80.0%)	140.8, 2C	10^–10^
Bulk Doping Only	Ta-05 [88]	enhanced electronic conductivity	2.5–4.8 ~ 2.0	279.0, 0.1C (1 C = 250 mA g^−1^)	213.2, 200 cycles, 1C (95.2%)	155.0, 5C	10^–13^
	1%Al [89]	decreased oxidation potential	2.0–4.8 ~ 2.0	305.0, 0.1C (1C = 250 mA g^−1^)	~ 192.3, 100 cycles, 1C (87.4%)	~ 220.0, 1C	–
	Al/Ti [90]	Al stabilizes lattice O, Ti expanding interlayer spacing	2.0–4.6 /	~ 270.0, 0.1C (1C = 200 mA g^−1^)	184.7, 500 cycles, 1C (89.7%)	~ 150.0, 5C	10^–14^
	Cd_0.03_S_0.03_ [91]	enhance diffusion kinetics	2.0–4.8 ~ 3.0	268.5, 0.1C (1 C = 250 mA g^−1^)	180.0, 200 cycles, 1C (84.4%)	153.0, 5C	1.4 × 10^–12^ after 150 cycles
	Gradient Al/Mg [92]	suppress oxygen release	2.0–4.6 ~ 3.5	~ 254.0, 0.1C (1C = 200 mA g^−1^)	150.0, 300 cycles, 1C (80.0%)	~ 120.0, 5C	10^–14^
Functionalized Multiscale Engineering	S, N co-doped carbon, oxygen vacancy gradients, and atomic-scale interface reconstructions [93]	oxygen vacancies and S, N co-doped carbon layer facilitated diffusion kinetics	2.0–4.8 /	292, 0.2C (1C = 250 mA g^−1^)	215.6, 500 cycles, 1C (86.6%)	190.0, 5C	10^–14^
	La^3+^, Zr^4+^ co-doped [94]	LaMnO_3+δ_/La_2_Zr_2_O_7_ coating facilitated diffusion kinetics, Zr expanding interlayer spacing)	2.0–4.8 ~ 1.2	~ 252, 0.1C (1 C = 250 mA g^−1^)	192.6, 200 cycles, 2C (75.1%)	~ 149.0, 5C	10^–14^
	Te^6+^ and Mg_3_(PO_4_)_2_ [95]	Te^6+^ enhances cationic redox, Mg_3_(PO_4_)_2_ coating and a cation-disordered interphase lower lattice-O activity	2.0–4.6 ~ 4.8	282.2, 0.1C (1C = 200 mA g^−1^)	222.2, 200 cycles, 1C (89.2%)	178.8, 10C	10^–10^
	Feox-2% [96]	oxygen vacancy and spinel phase facilitated diffusion kinetics	2.0–4.8 /	254.0, 0.1C (1 C = 250 mA g^−1^)	171.9, 300 cycles, 1C (86.4%)	155.1, 5C	–
	rocksalt domains coating and Al-doping [97]	Al stabilizes bulk lattice oxygen	2.0–4.8 /	248.8, 0.1C (1C = 250 mA g^−1^)	200.0, 200 cycles, 0.5C (93.6%)	120.5, 5C	10^–14^

#### Surface Modification

Surface modification strategies employing chemically stable and fast ion-conducting materials have been widely adopted to enhance interfacial Li^+^ transport and suppress parasitic reactions at the cathode-electrolyte interface. Oxide-based coatings, including LiNbO_3_, Li_4_Ti_5_O_12_, and Li_3_PO_4_, demonstrate the ability to facilitate Li^+^ diffusion across particle surfaces while mitigating electrolyte-induced degradation [83, 84]. The effectiveness of these coatings primarily depends on their lattice compatibility with the underlying cathode substrate. High crystallographic matching enables the formation of conformal, adherent protective layers, minimizes interfacial defects, and suppresses impedance buildup during repeated cycling. This atomic-scale registry between coating and substrate is essential for establishing uniform surface protection while maintaining long-term interfacial stability.

In addition to conventional oxide coatings, spinel-structured materials have emerged as promising surface modification agents, offering several advantages over traditional oxides like ZrO_2_, Al_2_O_3_, and AlF_3_. Spinel coatings provide three-dimensional Li⁺ transport channels with readily accessible tetrahedral 8a sites. This 3D transport framework not only improves rate capability but also stabilizes the surface layered structure by inhibiting oxygen evolution during cycling. For example, spinel-coated Li_2_MnO_3_ exhibits an extended O–O distance of 2.57 Å, compared to 1.40 Å in uncoated Li_2_MnO_3_, effectively reducing O_2_ formation and alleviating structural degradation. These improvements highlight the dual functionality of spinel coatings in simultaneously enhancing ionic transport and preserving structural integrity, rendering them particularly suitable for high-power applications [85].

Beyond oxide and spinel coatings, emerging strategies have explored polymer-based layers and hybrid composites to further enhance interfacial stability. Conductive polymers, such as polyaniline and polypyrrole, provide flexible and lightweight protective layers capable of accommodating volume changes during cycling. Hybrid coatings combining oxides (e.g., Al_2_O_3_ and ZrO_2_) with carbon-based materials like graphene, offer synergistic benefits by simultaneously providing both ionic and electronic conductivity [98]. Nevertheless, challenges remain in optimizing coating thickness, uniformity, and scalability for practical applications. Thin coatings in the range of 2–6 nm are preferred to minimize impedance while maintaining effective protection, but achieving uniform deposition on complex cathode morphologies remains technically demanding. Techniques including atomic layer deposition, chemical vapor deposition, and wet-chemical methods have been explored to address these challenges, with atomic layer deposition showing particular promise due to its precise control over thickness and conformality [99]. Moreover, the long-term stability of surface coatings under extreme conditions, such as high temperatures and aggressive electrolytes, requires further investigation to ensure compatibility with commercial battery systems.

#### Bulk Doping

In parallel with surface modifications, element doping into the bulk crystal lattice serves as an effective strategy for enhancing Li^+^ diffusion and reinforcing structural stability in LROs. The introduction of selective cations or anions species into the Li and TM layers, doping induces three distinct yet complementary mechanisms [88, 89, 100–102]. First, channel modulation is achieved by expanding the interlayer spacing and tuning the local geometric and topological environment of the Li^+^ diffusion pathways. This structural adjustment facilitates smoother Li^+^ transport by widening diffusion channels and alleviating geometric constraints along the migration pathway. Second, the stabilization originates from dopant-O orbital interactions that regulate the local O coordination/electronic structure and increase the oxygen vacancy formation energy (or suppress oxygen vacancy generation/propagation), thereby mitigating oxygen loss, surface reconstruction and TM migration, and thus alleviating irreversible phase transitions during cycling. Third, barrier engineering targets the energetic landscape of Li^+^ migration by lowering the activation energy via reduced electrostatic repulsion between Li^+^ and adjacent TM ions. While channel modulation focuses on the physical dimensions and connectivity of the migration pathway, barrier engineering directly addresses the energy required for Li^+^ to overcome transition states. A representative example is the dual-site Cd/S co-doping in Li_1.2_Ni_0.2_Mn_0.6_O_2_, where Cd^2+^ (ionic radius: 0.95 Å) incorporates into both Li and TM layers, and S^2−^ (1.84 Å) substitutes oxygen anions. This dual-doping strategy produces a pillar effect that maintains an expanded interlayer distance (4.83 Å) during deep delithiation, ensuring both structural openness and reduced Li^+^ hopping energy. As a result, the activation energy for Li^+^ diffusion is lowered, enabling a high-rate capability of 153.8 mAh g^−1^ at 5 C [91]. Moreover, the Li^+^ diffusion coefficient increases by nearly three times after Cd/S co-doping. This case illustrates how rational doping design can simultaneously optimize structural and kinetic properties, thereby significantly advancing the electrochemical performance of LROs.

#### Functionalized Multiscale Engineering

Although single modification strategies are effective in targeting specific properties of LROs, they often struggle to resolve the coupled and multiscale kinetic bottlenecks that govern Li^+^ transport, including bulk diffusion within the layered lattice, transport through grain boundaries and particle aggregates, and Li^+^ transfer across the cathode-electrolyte interphase. Conventional bulk doping and surface coating therefore often remain passive with respect to transport regulation. Bulk dopants can stabilize the layered framework yet may not prevent interfacial impedance growth, whereas coatings can suppress parasitic reactions but may introduce additional barriers to Li^+^ transport and still fail to suppress redox driven bulk and surface reconstruction. Moreover, for many reported dopant species and coating chemistries, performance benefits are frequently established in a largely empirical manner, while the underlying mechanistic origins remain insufficiently resolved. Improvements in rate capability or voltage retention are often attributed to broadly defined effects such as stabilized oxygen redox or suppressed interfacial reactions, yet the dominant kinetic contribution, whether arising from modified bulk Li^+^ migration topology, enhanced grain boundary conduction, or reduced interfacial charge transfer and ion transfer resistance, cannot always be unambiguously separated. This ambiguity is further compounded by the strong coupling among defect evolution, surface reconstruction, and CEI formation under high voltage cycling, implying that the same modification may operate through distinct mechanisms depending on composition and state of charge. Consequently, establishing transferable mechanistic descriptors that quantitatively link a given dopant or coating chemistry to Li^+^ transport kinetics remains a central challenge for predictive and rational design of LROs. These constraints have motivated the development of functionalized multiscale engineering strategies that co-regulate bulk and surface properties to optimize overall electrochemical performance, not merely by adding stabilizers, but by actively constructing and preserving continuous low-barrier Li^+^ pathways while regulating oxygen redox and suppressing transition metal migration [103].

One representative route is the construction of multifunctional protective layers that integrate surface passivation with subsurface defect and gradient engineering, commonly implemented via heteroatom-doped carbon coatings, oxygen vacancy gradients, and atomic-scale interface reconstructions [93]. In such designs, oxygen vacancies can reduce the activation energy for Li^+^ and transition metal migration by distorting local coordination environments and widening diffusion bottlenecks. At the same time, vacancy-mediated reconstruction can facilitate the formation of a coherent rocksalt like interphase that is structurally compatible with the underlying layered lattice. This coherency mitigates stress accumulation and suppresses crack propagation during cycling, thereby retarding impedance growth and preserving Li^+^ accessibility. Nevertheless, rational vacancy engineering requires fine control over vacancy concentration and spatial distribution to avoid adverse effects, consistent with the discussion in Sect. 2.1.2 [93].

A second functionalized paradigm involves site-programmed bulk and surface-differentiated co-doping to build phase connected transport networks at mesoscopic length scales. For example, co-doping with La^3+^ and Zr^4+^ can generate a triple phase interface composed of LaMnO_3+*δ*_ and La_2_Zr_2_O_7_, which exhibits high ionic conductivity and facilitates efficient charge transport along grain boundaries [94]. In this configuration, La^3+^ predominantly segregates toward the particle surface to tailor interfacial structure and suppress electrolyte decomposition, whereas Zr^4+^ incorporates into the crystal lattice to enhance bulk stability. Such differentiated doping can also expand interlayer spacing and improve Li^+^ diffusion pathways, leading to higher diffusion coefficients and superior rate performance over a broad state of charge window.

To further stabilize anionic redox while maintaining transport continuity, redox transport co-engineering combines bulk redox regulation with Li^+^ permeable surface chemistry. Dopants such as Mg^2+^, Al^3+^, or Te^6+^ can promote flexible TM-O coordination or reduce the average transition metal valence, thereby stabilizing anionic redox activity and mitigating redox driven structural blocking of Li^+^ channels [95, 104]. Such bulk modifications are often coupled with phosphate-based surface coatings, for example, Mg_3_(PO_4_)_2_, which resist acidic attack while maintaining facile Li^+^ conduction, ensuring that highly active redox centers remain electrochemically accessible without triggering parasitic interfacial reactions. Overall, these multicomponent approaches exemplify how doping and coating become qualitatively different once functionalized, shifting from additive protection to engineered defect topology, phase connectivity, and adaptive interphases that co-regulate lattice stability, interface chemistry, and Li^+^ transport kinetics to deliver improved rate capability, mitigated voltage decay, and prolonged cycling stability.

### Particle Morphology Design

**Fig. 6:**
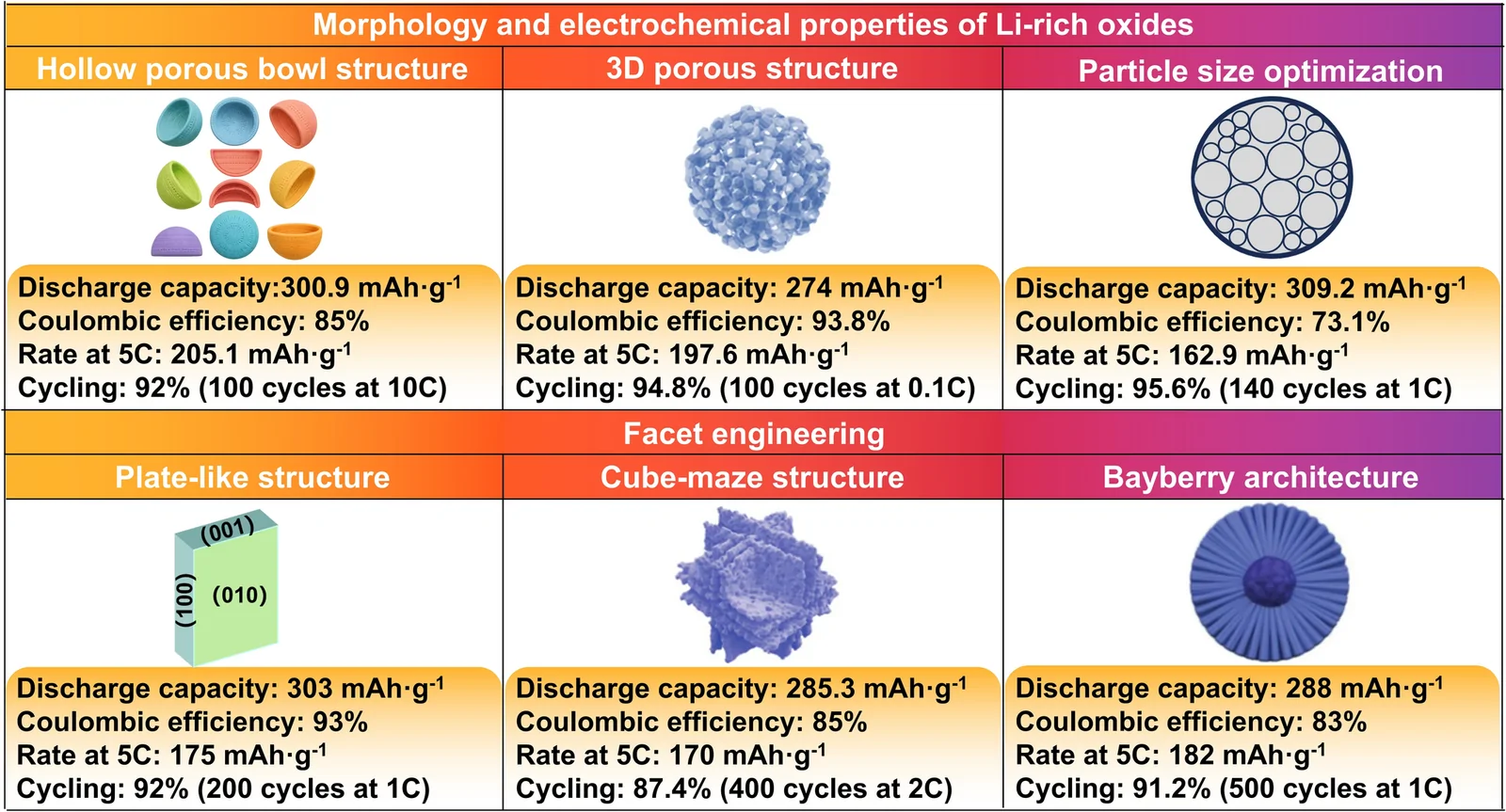
Various particle morphology design of LROs [106–111]

The kinetics of Li^+^ transport are influenced by particle morphology, which also affects stress distribution and structural stability during cycling. Various morphological designs, ranging from hollow spheres and microrods to hierarchically assembled polycrystalline structures, have been extensively explored to enhance electrochemical performance [105]. Zhang et al. [106] uses bowl-shaped carbonaceous particles as the predominant template and polyvinylpyrrolidone as a soft template, with a carefully controlled heating rate to achieving the desired morphology. This unique architecture combines the advantages of hollow structures with enhanced particle packing density, resulting in cathodes that deliver high discharge capacities of 300.9 mAh g^−1^ at 0.1 C, 248.5 mAh g^−1^ at 1 C, 164.5 mAh g^−1^ at 10 C, and maintains 103.6 mAh g^−1^ even at a high rate of 20 C. Similarly, Liu et al. [107] demonstrated that a CO_2_-based chemical delithiation strategy could successfully construct a 3D nanoporous structure in Li-rich layered oxides. Nanoporous structure not only facilitates ion diffusion but also maintains a high tap density, improving both rate capability and volumetric energy density. Controlling particle architecture across different length scales, from primary to secondary particles, is essential for enhancing ion and electron transport, minimizing interfacial side reactions, and preserving structural integrity during cycling (Fig. 6).

Furthermore, particle size optimization is a critical strategy for enhancing Li^+^ transport kinetics in cathode materials. A smaller particle size reduces the solid-state diffusion path length for Li^+^ ions, which directly improves rate capability [17]. In a study by Fang et al. [110], samples synthesized from different manganese precursors were compared. The LROs prepared using Mn(NO_3_)_2_·4H_2_O (denoted as N-LMR) exhibited the smallest average particle size of 170 nm and the narrowest size distribution. In contrast, samples derived from Mn(CH_3_CO_2_)_2_·4H_2_O and MnCO_3_ showed significantly larger average particle sizes of about 290 nm. The refined particle size of N-LMR facilitates faster Li^+^ extraction and insertion, resulting in a high initial discharge capacity of 309.2 mAh g^−1^ at 0.1 C and 208.1 mAh g^−1^ at 1 C. The corresponding reduction in polarization and the enhanced cycling stability, with 95.6% capacity retention after 140 cycles at 1 C, further corroborate the beneficial effect of particle size minimization. These results unequivocally demonstrate that optimizing particle size is an effective approach to simultaneously optimize Li^+^ transport, enhance structural integrity, and extend the cycle life of LROs.

Although nano- or porous morphologies have been widely adopted to shorten Li^+^ diffusion paths and enhance electrode–electrolyte contact, their high specific surface area tends to promote side reactions and accelerate the formation of a thick CEI layer, resulting in low Coulombic efficiency and considerable irreversible lithium loss. In contrast, monodispersed micrometer-sized single-crystal particles represent a promising alternative, offering superior structural stability, higher packing density, and suppressed parasitic reactions due to their low external surface area and absence of internal grain boundaries [112]. An ingenious design for such microstructures was demonstrated by Xia et al. [113] through the synthesis of Li_1.2_Ni_0.13_Co_0.13_Mn_0.54_O_2_ particles incorporating twin structures that serve as “bridges” interconnecting distinct Li^+^ diffusion tunnels. This design simultaneously enhances the Li⁺ diffusion coefficient and maintains structural integration, delivering a high specific capacity of 253 mAh g^−1^ at 1 C along with 85% capacity retention and 94.2% voltage retention after 200 cycles.

The crystallographic orientation of exposed surfaces is one of important roles in Li^+^ diffusion kinetics. While this effect is inherently coupled with grain boundary structures and surface chemistry, recent studies have shown that certain orientations can act as fast Li^+^ transport pathways. In conventional layered LROs, Li^+^ preferentially migrates along the {010} planes via 2D diffusion channels within the Li slabs, whereas the (001) planes pose considerable resistance due to densely packed TM-O layers. As such, engineering crystal growth conditions to promote {010} surface exposure has proven effective in improving ionic mobility by minimizing its surface energy relative to other facets [108]. Morphologies favoring {010} orientation, such as platelike structures [109] or radially arranged spherical [111], can enhance Li^+^ transport kinetics, alleviate local stress accumulation, and improve both rate capability and cycling stability [114]. A representative example of an advanced morphology is the bayberry-like superstructure, which comprises a dense spherical core of agglomerated nanoparticles enveloped by radially aligned nanorods possessing exposed (003) facets [111]. This hierarchical structure facilitates multidirectional transport of both Li^+^ and electrons, and homogenized internal stress distribution during cycling. As a result, this design significantly reduces charge transfer resistance (*R*_ct_ = 158.3 Ω) and improves Li^+^ diffusion coefficients (${D}_{{\mathrm{Li}}^{+}}$=5.96 × 10^–16^ cm^2^ s^−1^), reflecting enhanced transport reversibility and long-term structural durability.

Overall, particle morphology regulates Li^+^ transport kinetics in LROs by governing transport connectivity, interfacial accessibility, and stress evolution during cycling, rather than merely shortening diffusion distance. Hierarchical, hollow, and nanoporous morphologies create multidirectional Li^+^ pathways and improve electrolyte penetration, enhancing kinetics but also increasing electrochemically active surface area and thereby intensifying surface-driven side reactions and CEI formation. In contrast, compact morphologies such as single-crystalline or twin-structured particles suppress parasitic reactions by eliminating internal grain boundaries while maintaining continuous Li^+^ diffusion networks. Morphology designs that combine interconnected transport pathways with preferential exposure of fast ion-conducting facets, such as {010}, enable fast kinetics without excessive surface exposure. These insights indicate that fast-kinetic LROs should be designed through morphology-enabled control of Li^+^ transport and interfacial reactivity, rather than relying solely on surface area maximization.

### Bulk Structural Optimization

While surface modifications offer specific performance enhancements, optimizing the bulk structure of LROs is the key point for achieving substantial improvements in Li^+^ diffusion kinetics. Addressing the challenge of limited Li^+^ transport within the bulk structure requires strategies such as the introduction of oxygen vacancies to facilitate Li^+^ migration, the incorporation of spinel phases to leverage their superior 3D diffusion pathways, and the rational regulation of calcination conditions to engineer optimal microstructures.

#### Vacancy Engineering

The introduction of oxygen vacancies into the crystal lattice of LROs is an effective strategy for enhancing Li^+^ transport by creating additional migration pathways and lowering energy barriers [115]. Density functional theory (DFT) calculations confirm that such vacancies can reduce the migration energy barrier to as low as 0–0.21 eV, significantly enhances ion mobility, and thereby improves both rate capability and cycling stability [116]. Beyond enhancing ionic transport, oxygen vacancies also help suppress irreversible oxygen release from the lattice, thereby maintaining the structural integrity of the layered framework. By precisely controlling the concentration and distribution of oxygen vacancies, it is possible to optimize Li^+^ diffusion without compromising the bulk structure, leading to improved electrochemical performance and mitigated voltage decay during long-term cycling.

**Fig. 7:**
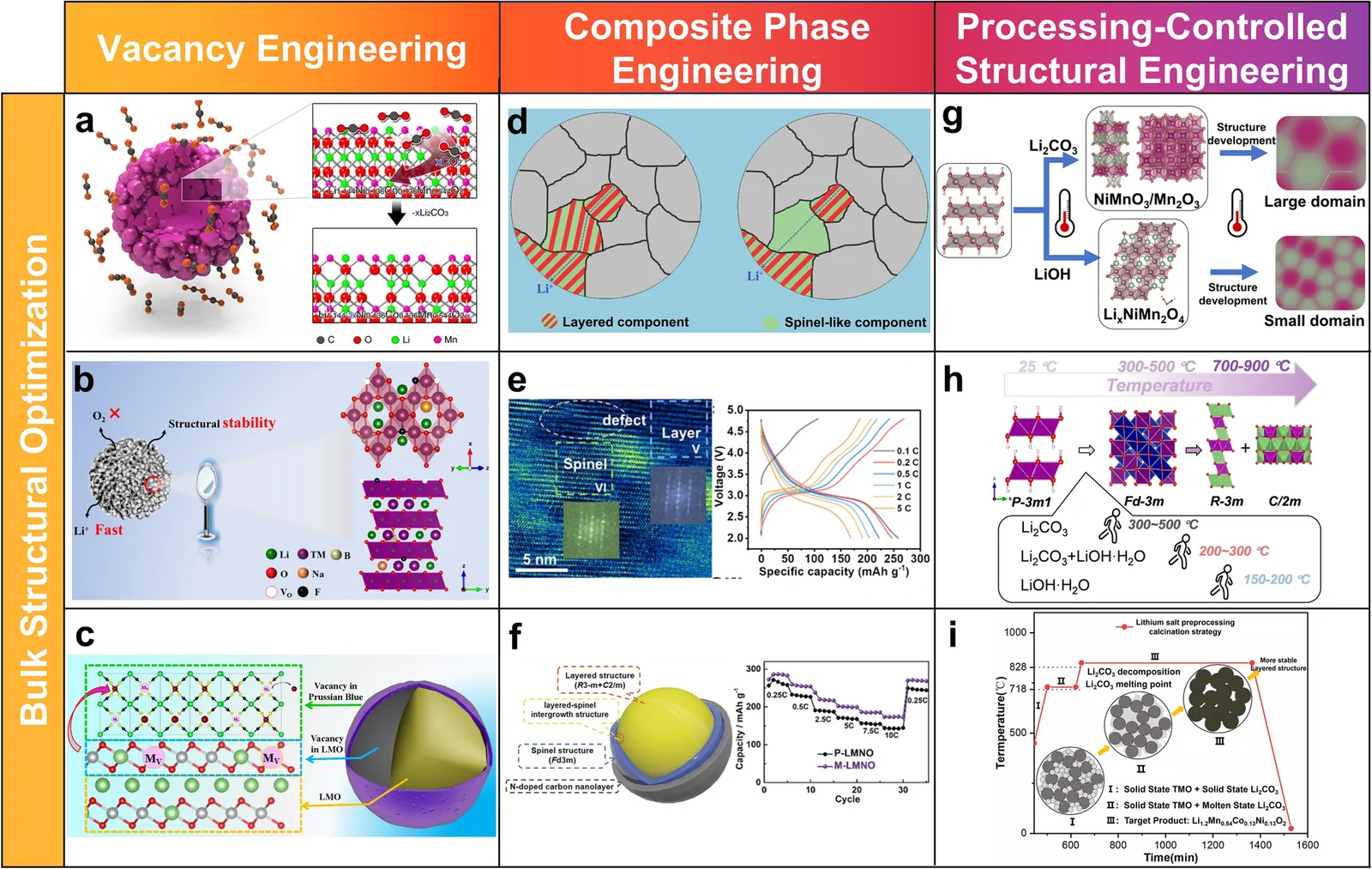
**a** Gas–solid interfacial modification [116]. **b** A multifunctional interface layer comprising a spinel phase with oxygen vacancies and Na/B/F triple-doping [118]. **c** Prussian blue coating featuring manganese vacancies [121]. **d** Schematic illustration for Li-ion diffusions within the spinel-layered structure [122]. **e** Layered-spinel intergrown Li-Mn-O structure [123]. **f** Integrated surface treatment for enhanced lithium-ion diffusion: oxygen vacancies, spinel phase, and N-doped carbon nanolayers [124]. **g** Lithium source-dependent synthesis pathways and divergent microstructure in LROs [125]. **h** Role of lithium sources (LiOH, Li_2_CO_3_, and their mixtures) in the synthesis process [126]. **i** Li_2_CO_3_ preprocessing calcination strategy [127]

Several strategies have been established to controllably introduce oxygen vacancies into LROs while preserving the overall crystallinity. Among them, gas–solid interfacial reactions using mild agents like (NH_4_)_3_PO_4_ or NH_4_Cl can selectively create a 10–20 nm defective surface layer (Fig. 7a), enhancing near-surface Li^+^ transport without altering the bulk phase [116]. Alternatively, thermal annealing in inert or weakly reducing atmospheres generates oxygen vacancies throughout the bulk lattice while largely retaining its long-range structural order [117, 118]. Peng et al. [118] further optimized this approach via a two-step process involving liquid NaBF_4_ treatment and in situ reactions during sintering, which introduced oxygen vacancies, spinel domains, and multielement doping (Na, B, F). This modification enhances conductivity and stabilizes the oxygen lattice, yielding a material with higher operating voltage and 89.94% capacity retention after 100 cycles at 1 C (Fig. 7b). Aliovalent doping represents another effective route, such as Na incorporation via molten-salt templating, generating charge-compensating oxygen vacancies while concurrently enabling particle morphology [119]. These bulk and surface modifications serve to broaden Li^+^ diffusion pathways, suppress oxygen release, and inhibit surface reconstruction. This synergistic action ultimately leads to improved initial capacity, reversibility, and structural stability [120].

In contrast, another approach involves the introduction of Mn vacancies, as demonstrated by Li et al. [121], who successfully incorporated interfacial Mn vacancies into a composite of 0.5Li_2_MnO_3_·0.5Li[Ni_1/3_Co_1/3_Mn_1/3_]O_2_, as shown in Fig. 7c. The Mn vacancies not only enhance the durability of the Prussian blue coating layer but also activate the composite, improving reversibility at both high and low current rates. Furthermore, the Mn vacancies promote the transformation of the composite into an amorphous phase with improved kinetics, showcasing the potential of Mn vacancy engineering to further enhance performance.

In summary, these approaches demonstrate that rational design and precise control of oxygen/Mn vacancy formation, whether localized at the particle surface or distributed throughout the lattice, can substantially enhance ionic transport kinetics in LROs. Through the synergistic integration of surface and bulk vacancy engineering, it becomes feasible to simultaneously achieve accelerated Li^+^ diffusion, mitigated voltage decay, and prolonged cycling stability.

#### Composite Phase Engineering

Single-phase cathode materials face an inherent compromise: Layered oxides offer high energy density but limited two-dimensional Li^+^ diffusion, while spinel structures provide rapid three-dimensional Li^+^ transport at the cost of lower capacity (~ 130 mAh g^−1^). Composite phase engineering, which synergistically integrates both phases, has been demonstrated as an effective strategy to overcome these limitations. This integrated architecture harnesses the high capacity of layered oxides and the fast ionic conduction of spinel, effectively optimizing both energy density and rate capability [128, 129].

Recent advancements in synthesis methods have facilitated the formation of homogeneous layered-spinel composites with well-distributed spinel and layered domains. For instance, solvothermal precursor methods coupled with controlled calcination yields spherical secondary particles with uniform phase distribution. This structural design significantly enhances Li^+^ transport, as the ions now follow more direct pathways through the spinel regions rather than navigating complex paths between discrete particles. As illustrated in Fig. 7d [122], this direct conduction path allows for faster ion migration, reducing the overall transport resistance. The resulting materials exhibit high Li^+^ diffusion coefficients (around 2.5 × 10^–12^ cm^2^ s^−1^), along with high retained capacities exceeding 185 mAh g^−1^, even at high current densities (e.g., 1200 mA g^−1^). A more refined approach involves constructing an layered-spinel intergrown Li-Mn-O structure via an ion exchange process, typically by reacting Na_2_Mn_3_O_7_ with lithium salt under optimized calcination conditions, as shown in Fig. 7e [123]. The resulting intergrown composite exhibits expanded ion channels, which facilitate improved Li⁺ migration and lead to remarkable high-rate performance (169.2 mAh g^−1^ at 5 C), surpassing that of previously reported Li-rich manganese-based cathode materials. In addition to this structural enhancement, surface engineering plays a crucial role in optimizing performance. A simple, one-step treatment using urea can simultaneously induce oxygen vacancies, transform the surface layer into a spinel-layered heterostructure, and apply a protective N-doped carbon coating, as shown in Fig. 7f [124]. This three-in-one treatment has demonstrated impressive results, including a discharge capacity of 253.5 mAh g^−1^ and a capacity retention of 89.9% after 500 cycles, further highlighting the potential of composite phase engineering.

In summary, the integration of spinel and layered domains within composite materials significantly improves both Li^+^ transport and electrochemical performance. The spinel phases provide rapid Li^+^ conduction pathways, while the layered regions contribute to high capacity and voltage stability. Through the careful design of these structural motifs, including bulk and surface modifications, composite phase engineering not only enhances rate capability and cycling stability but also overcomes the traditional trade-off between energy and power density. This strategy positions layered-spinel composites as ideal candidates for the development of high-performance, next-generation LIBs, offering a promising solution for meeting the increasing demands of advanced energy storage systems.

#### Processing-Controlled Structural Engineering

The calcination process and the choice of lithium precursor play a decisive role in determining the crystallographic ordering, domain architecture, and electrochemical performance of LROs. Calcination directly governs the nucleation and growth of the Li_2_MnO_3_ phase, the distribution of TM cations, and the stability of the layered framework [125]. Key parameters including temperature, duration, and atmospheric environment must be carefully optimized, because prolonged exposure to elevated temperatures can induce excessive grain growth and cation migration [130]. These effects destabilize the layered structure, promote unwanted phase transformations, and accelerate voltage fade during extended cycling. In addition, the choice of lithium precursor strongly influences the microstructural evolution of Li_2_MnO_3_. Lithium carbonate generally requires higher calcination temperatures, which tend to produce coarse and aggregated Li_2_MnO_3_ domains. These large domains contribute to compositional heterogeneity and exhibit great susceptiblility to oxygen release and structural degradation during cycling. By contrast, lithium hydroxide monohydrate facilitates Li at lower temperatures, yielding smaller and more evenly dispersed Li_2_MnO_3_ domains within a homogeneously distributed transition metal matrix. This fine domain architecture improves lattice coherence, enhances the reversibility of anionic redox processes, and suppresses voltage decay, thereby promoting superior structural and electrochemical stability. A schematic representation of the overall solid-state synthesis pathways, illustrating the interplay between processing conditions and structural evolution, is also shown in Fig. 7g–i.

Beyond calcination and precursor selection, several other synthesis-related factors also critically influence the crystal structure, defect distribution, and particle size of LROs. These factors include precursor molar ratios, the uniformity of particle mixing, the morphology of initial precursors, and the incorporation of fluxing agents or molten salts [126, 127]. Precise regulation of these parameters enables tuning of the bulk phase composition and optimization of the domain architecture, which are essential for achieving robust structural stability and superior Li⁺ transport kinetics. For example, the use of molten salts can accelerate diffusion processes and facilitate homogeneous integration of layered and spinel domains, while carefully adjusted precursor ratios prevent phase segregation and compositional heterogeneity. Collectively, these synthetic strategies provide a powerful toolbox for engineering LROs with controlled microstructures and improved electrochemical performance.

### Redox Chemistry Engineering

The activation of lattice oxygen redox in LROs presents a dual-edged sword: on the one hand, it enables unprecedented capacity enhancement beyond conventional TM redox limits. On the other hand, it inevitably introduces challenges such as sluggish kinetics, severe voltage hysteresis, and lattice instability under prolonged cycling. At high states of delithiation, the participation of oxygen in charge compensation can trigger irreversible oxygen release, phase transitions, and TM migration, collectively undermining electrochemical reversibility [56]. These issues stem from the intrinsically unstable nature of anionic redox, which operates at higher potentials and couples strongly to the lattice environment. Therefore, engineering strategies that stabilize lattice oxygen while promoting reversible redox activity have emerged as a critical direction in the pursuit of high-energy-density cathodes. From a transport perspective, the slow electron/ion-coupled charge compensation during oxygen redox elevates kinetic polarization and charge transfer resistance at high voltages, which can markedly depress the apparent Li^+^ diffusion during the activation window even when the Li layer spacing is expanded.

A highly effective strategy involves the incorporation of redox-active transition metals with strong M–O covalency (e.g., Co and Ni), which can rebalance charge compensation and accelerate coupled electron/Li^+^ transfer [131–133]. Among these, cobalt plays a particularly crucial role, which is attributed to its pronounced Co–O covalency and the capability for rapid, reversible valence transition. The Co^(3+*δ*)+^/Co^4+^ redox couple not only facilitates partial delithiation before the onset of complete TM oxidation but also lowers the activation barrier for O^2−^/O_2_^−^ redox processes [37, 134]. Importantly, this “fast cationic buffering” can alleviate interfacial polarization and impedance buildup at high states of charge, thereby helping sustain effective Li^+^ transport under high-rate operation. In parallel, Ni^2+^/Ni^4+^ redox provides an kinetically facile cationic pathway that can tune the kinetics of oxygen redox, mitigate voltage hysteresis, and inhibit TM migration. Mechanistically, Ni and Co do not play identical roles in regulating anionic redox: Co tends to enhance ligand-to-metal charge transfer and accelerate oxygen redox kinetics, whereas Ni is more effective in suppressing oxygen release and surface reconstruction, for example via the formation of a Ni-enriched rocksalt-like passivation layer, leading to improved redox reversibility and a slower rise in impedance [135]. These distinctions suggest that optimizing the Ni/Co ratio, rather than increasing the single TM content, enables the simultaneous improvement of redox kinetics and the retention of fast Li^+^ transport. Notably, stronger covalency and higher Ni/Co contents may also increase surface reactivity toward electrolyte decomposition and promote thicker CEI formation, which can offset intrinsic transport benefits by increasing interfacial resistance [58]. Therefore, redox chemistry engineering is often most effective when coupled with surface stabilization strategies (e.g., coatings or electrolyte additives) to sustain low charge transfer resistance at high potentials.

In parallel with transition metal mediation, anion substitution has been established as a potent complementary strategy for modulating the redox behavior of LROs [136, 137]. The partial substitution of oxygen with anions of differing electronegativity (e.g., F^−^ or S^2−^) directly perturbs the electronic structure and local coordination environment, thereby effectively tuning the overall redox activity [138]. For example, sulfur doping reduces the average operating voltage but enhances both electronic conductivity and Li^+^ diffusivity. In contrast, fluorine doping strengthens the transition metal-anion bonding, which improves structural stability and suppresses oxygen loss. Although S-doped systems often exhibit reduced theoretical energy density, co-substitution with halides or chalcogenides has been demonstrated to restore high operating potentials while preserving favorable kinetics. These tailored anionic substitutions mitigate voltage hysteresis, suppress lattice oxygen instability, and ultimately provide an optimal compromise between energy density and structural integrity. By stabilizing high-voltage redox activity and restraining surface reconstruction, anion substitution can also help maintain low interfacial impedance and thus improve effective Li^+^ transport under practical rates.

In summary, transition metal-mediated redox catalysis and targeted anion substitution represent two complementary strategies for stabilizing lattice oxygen redox in LROs. The former mitigates kinetic barriers and facilitates reversible oxygen redox activity, whereas the latter tunes the local coordination environment and restrains irreversible structural evolution. When synergistically integrated, these strategies work in concert to harmonize cationic and anionic redox reactions and inhibit oxygen loss, thereby preserving the structural integrity of the layered framework. Crucially, by suppressing TM migration that blocks Li layers and by limiting impedance growth at high potentials, redox chemistry engineering translates directly into more robust Li^+^ transport and improved rate capability.

## Applications of Advanced Characterization Techniques

**Fig. 8:**
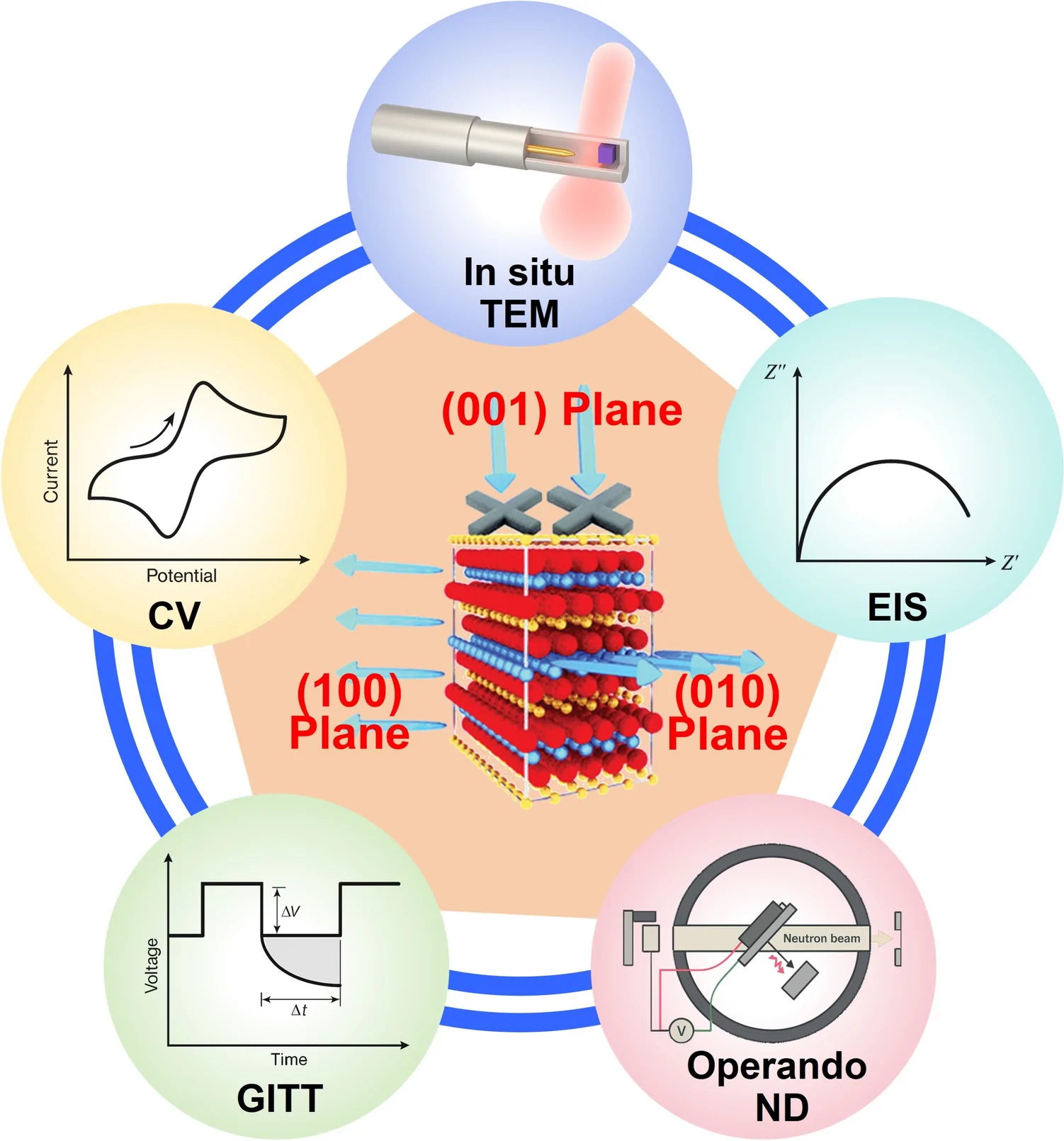
Schematic of characterization techniques for LROs [140–145]

Understanding the transport dynamics of Li^+^ in LIBs remains challenging due to the ultrafast kinetics and spatially heterogeneous behavior. As Li^+^ migrate across bulk crystal structures, grain boundaries, and electrode–electrolyte interfaces, they encounter dynamically evolving electrochemical environment. Conventional ex situ and static characterization techniques fail to capture these time-sensitive processes, resulting in an incomplete understanding of ionic transport pathways and reaction mechanisms. For example, recent ex situ nuclear magnetic resonance (NMR) studies have revealed limited Li^+^ reinsertion into TM layers, suggesting that reversible migration within octahedral sites may be highly restricted during cycling [139, 140]. These findings highlight the urgent need for advanced in situ and operando techniques capable of resolving Li^+^ dynamics with high temporal and spatial resolution across multiple interfaces and phases (e.g., electrochemical impedance spectroscopy (EIS), cyclic voltammetry (CV), GITT, and operando neutron diffraction (ND), in situ transmission electron microscopy (TEM), as illustrated in Fig. 8. For clarity, a concise comparison of the key Li^+^ transport information accessible by these techniques, together with their characteristic time and length scales and experimental limitations, is summarized in Table 3.

**Table 3:** Comparison of electrochemical and structural characterization techniques for probing Li^+^ transport in LROs

Technique	Li^+^ transport information obtained	Time scale	Length scale	Limitations
CV	Apparent Li^+^ diffusion coefficient, redox reversibility, kinetic polarization	s-min	Electrode	Assumes semi-infinite diffusion; convoluted surface and bulk contributions
EIS	Charge transfer resistance, interfacial impedance, Li^+^ diffusion via Warburg analysis	ms-s	Bulk/interface	Model dependence, overlapping processes require advanced analysis (DRT)
GITT	Chemical diffusion coefficient as a function of state of charge	min-h	Particle	Long testing time, sensitive to experimental parameters
Operando ND	Li site occupancy, phase transitions, bulk Li⁺ migration pathways	min-h	Bulk crystal	Low neutron flux, complex cell design, limited temporal resolution
In situ TEM	Direct visualization of Li^+^ diffusion, TM migration, and interfacial degradation	ms-s	Atomic to nanoscale	Beam damage, limited field of view, non-representative environments

Emerging researches now integrate electrochemical measurements, structural probes, and theoretical simulations into unified platforms. Notably, the combination of time- and space-resolved operando characterization techniques with DFT and kinetic modeling, offer a powerful route to decode the fundamental mechanisms governing ion migration, interfacial evolution, and degradation in LROs. The following sections survey key representative electrochemical and structural characterization techniques that have proven indispensable for probing these mechanisms across multiple scales, from microscopic processes and device-level performance.

### Electrochemical Analysis Methods

Electrochemical techniques capable of resolving Li^+^ transport in real-time have become indispensable tools for LIBs. Among them, CV remains one of the most versatile and widely used methods for probing redox behavior, interfacial kinetics, and diffusion limitations in electrode materials. By applying a linearly varying voltage and recording the resulting current response, CV provides access to key kinetic parameters such as electron transfer coefficients, redox potentials, and peak separation. Importantly, the Li^+^ diffusion coefficient (${D}_{{Li}^{+}}$) can be derived using the Randles–Sevcik equation:

1 $$\begin{array}{*{20}c} {i_{p} = 0.4463n{\mathrm{FAC}}\left( {\frac{{nFvD_{{Li^{ + } }} }}{{{\mathrm{RT}}}}} \right)^{\frac{1}{2}} } \\ \end{array}$$

where ${i}_{p}$ represents the peak current, *n* is the number of electrons transferred, *A* is the electrode area, *C* is the ion concentration, *F* is the Faraday constant, *R* is the gas constant, T is the temperature, *v* is the scan rate, and ${D}_{{\mathrm{Li}}^{+}}$ is the diffusion coefficient.

EIS complements CV by providing a frequency-domain perspective on interfacial and bulk transport processes. EIS measures the complex impedance of a system in response to a small-amplitude AC perturbation, yielding information on charge transfer resistance, double-layer capacitance, ionic diffusion, and CEI behavior. In the context of LROs, EIS is particularly useful for tracking the evolution of surface films and internal resistance induced by oxygen loss or TM migration. For instance, surface coatings such as polyaniline (PANI) have been shown to significantly reduce the surface film resistance (from 64.52 to 7.15 Ω over 10 cycles), enhancing Li^+^ mobility and mitigating interface degradation [146]. The diffusion coefficient ${D}_{{\mathrm{Li}}^{+}}$ can also be estimated from the Warburg impedance using the following relation:

2 $$\begin{array}{*{20}c} {D_{{{\mathrm{Li}}^{ + } }} = \frac{{0.5R^{2} T^{2} }}{{A^{2} n^{4} F^{4} C^{2} \sigma^{2} }}} \\ \end{array}$$

where *σ* is the Warburg coefficient determined from the low-frequency linear region of the Nyquist plot.

While equivalent circuit models are commonly used to fit EIS spectra, they often oversimplify real battery systems where multiple overlapping processes coexist. To address this, the distribution of relaxation times (DRT) method has been introduced as a model-free approach that transforms impedance data into discrete relaxation events as a function of time. DRT enables resolution of fast (e.g., surface charge transfer) and slow (e.g., bulk diffusion) processes, providing clearer attribution of resistance origins [147]. For example, in uncoated LROs, DRT analysis reveals multiple overlapping interfacial processes with relaxation times ranging from 10 to 1000 ms, and increasing total resistance (~ 7000 Ω) after prolonged cycling. Surface-engineered samples, by contrast, display significantly lower DRT peak intensities and final resistance (~ 300 Ω), indicating enhanced interfacial stability and kinetics [148].

The GITT offers a time-domain analysis of Li^+^ intercalation kinetics by applying a series of current pulses followed by rest periods. By analyzing the voltage response during and after each pulse, GITT provides the chemical diffusion coefficient as a function of state of charge [149]. The corresponding equation is:

3 $$\begin{array}{*{20}c} {D_{{Li^{ + } }} = \frac{4}{\pi \tau }\left( {\frac{{m_{B} V_{m} }}{{M_{B} S}}} \right)^{2} \left( {\frac{{\Delta E_{s} }}{{\Delta E_{\tau } }}} \right)^{2} \left( {\tau \ll L^{2} /D_{{Li^{ + } }} } \right)} \\ \end{array}$$

where *τ* is the pulse duration, *m*_*B*_, *M*_*B*_, and *V*_*m*_ are the mass, molar mass, molar volume of active material, and *S* is the electrode area, and Δ*E*_*τ*_ and Δ*E*_*s*_ are the transient and steady-state voltage changes, respectively. GITT has been particularly effective in revealing state-dependent diffusion behaviors. For example, a dramatic drop in lg*(*${D}_{{\mathrm{Li}}^{+}}$) from −12.8 to − 17 has been observed as the voltage increases from 4.3 to 4.5 V, highlighting kinetic sluggishness at high states of delithiation, as shown in Fig. 3h [60].

In summary, CV, EIS, and GITT each provide valuable insights into Li^+^ transport but have distinct limitations, as outlined in Table 3. CV offers high temporal resolution for rapid kinetic measurements, though it assumes semi-infinite diffusion, potentially overlooking surface and bulk effects. EIS excels in frequency-domain analysis of interfacial and bulk processes but can oversimplify complex systems with overlapping resistive behaviors. GITT reveals state-dependent diffusion but is sensitive to experimental parameters like pulse duration. A more effective strategy would combine these techniques, using CV for initial kinetic screening, EIS for interfacial process analysis, and GITT for state-dependent diffusion. Integrating methods like the DRT with EIS can further enhance the clarity of complex resistive processes.

### In Situ Structural Characterization Techniques

**Fig. 9:**
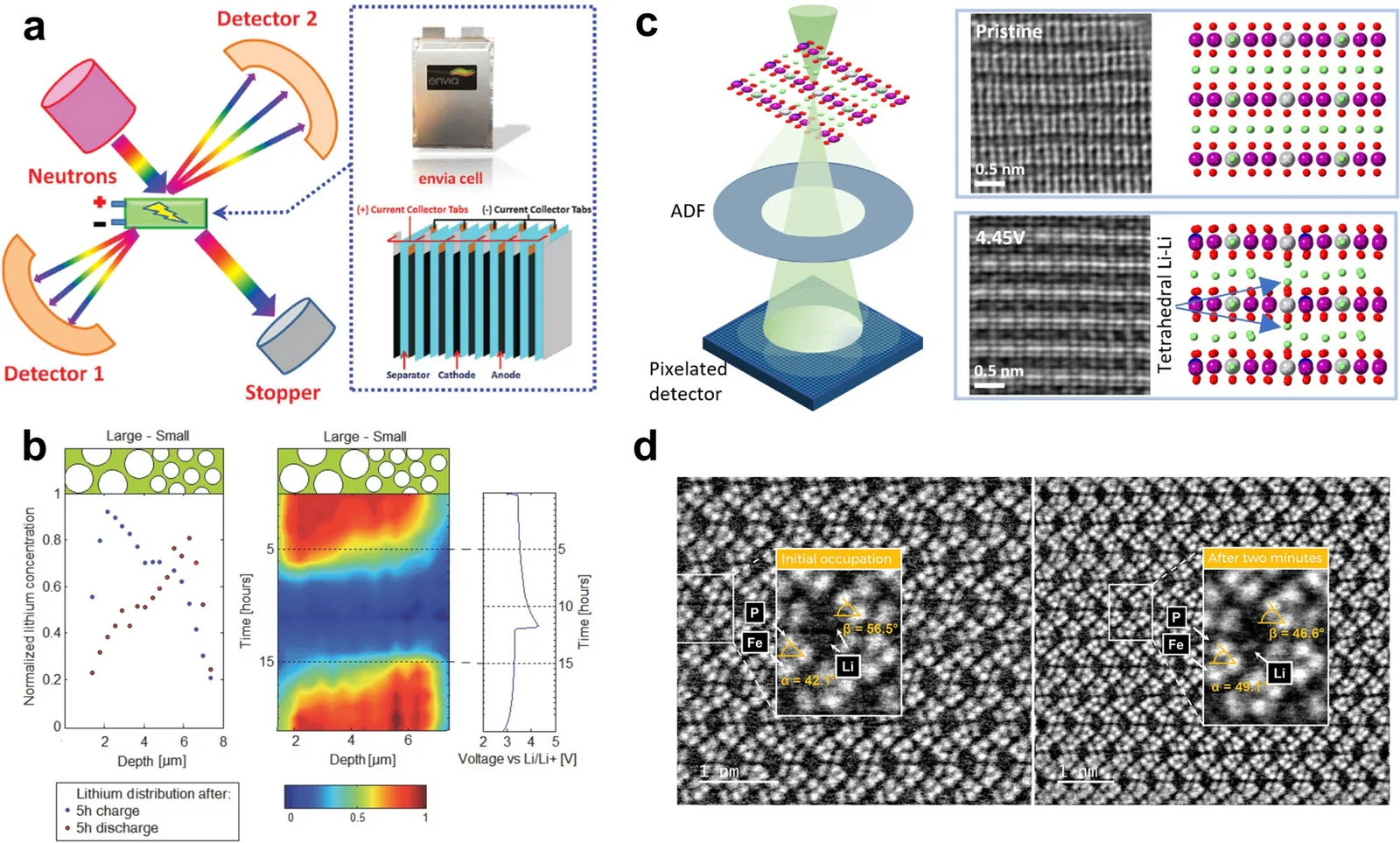
**a** Schematic of an operando ND experimental set-up based on the LROs/Si pouch cell [82]. **b** In situ ND spectra of LiFePO_4_ materials during C/10 cycling [153]. **c** Focused-probe electron ptychography with synchronized ADF imaging on Li_1.2_Ni_0.13_Mn_0.54_Co_0.13_O_2_ [55]. **d** Direct observation of atom column distortion as a consequence of local Li-diffusion and Li-distribution in individual channels over time [157]

Beyond electrochemical methods, structural probes capable of tracking atomic-scale transformations in real time are essential for deciphering Li^+^ transport mechanisms. Operando ND is especially valuable due to its deep penetration, sensitivity to light elements such as Li and O, and ability to distinguish transition metals via their unique scattering lengths (e.g., Li: −1.90 fm, O: 5.803 fm, Ni: 10.3 fm, Mn: −3.73 fm, Co: 2.49 fm), as illustrated in Fig. 9a, b [150]. From a transport perspective, operando ND enables quantitative refinement of (i) Li site occupancies, (ii) Li/TM antisite mixing, and (iii) lattice parameters/interlayer spacing as a function of state of charge, structural descriptors that directly regulate the availability and continuity of Li^+^ migration pathways in layered oxides. ND has been used to identify phase transitions, Li site occupancies, and local distortions in LROs. Nevertheless, its application is limited by low neutron flux, long data acquisition times, and interference from hydrogen-containing components. Mitigation strategies include using deuterated electrolytes, silicon-based cell casings, and large-format pouch cells (10–100 times volume of typical operando X-ray diffraction (XRD) cells) to improve signal-to-noise ratios [82, 151–155]. Notably, recent operando ND advances on layered Li-rich cathodes demonstrate that time-resolved tracking of phase evolution together with refined site occupancy and disordering parameters can be directly linked to transport degradation, where increased cation disordering and irreversible Li/TM migration are associated with hindered Li^+^ percolation and growing polarization during cycling [156]. Therefore, rather than directly observing Li^+^ migration trajectories, operando ND should be viewed as providing time-resolved, site-sensitive structural metrics that constrain and explain Li^+^ transport limitations under practical cycling conditions.

In situ TEM provides direct observation of atomic-scale structural and chemical evolution in operating batteries, capturing dynamic processes such as Li^+^ diffusion, TM migration, and interfacial degradation [157]. Recent advances, including chip-based cells and aberration-corrected optics, now enable operando tracking of crystallographic transformations under applied potential. For instance, single-crystal LiCoO_2_ has been observed transforming into nanocrystalline domains at high voltages, revealing dislocation-driven Li^+^ pathways [158]. More recently, focused-probe electron ptychography combined with annular dark-field (ADF) imaging and advanced phase reconstruction has captured sub-angstrom Li^+^ dynamics in Li-rich layered cathodes, as shown in Fig. 9c [55]. The emergence of tetrahedral Li–Li dumbbells at ~ 4.45 V and their subsequent disappearance upon TM migration illustrate a local breakdown of the continuous o-t-o Li-diffusion network in the Li layers. At the macroscopic level, the progressive blocking and rerouting of Li^+^ percolation pathways manifest as increased polarization and sluggish Li^+^ transport, leading to incomplete (de)lithiation, loss of accessible high-voltage capacity, and a gradual shift of the discharge profile toward lower potentials, ultimately resulting in voltage fade and kinetic deterioration of LROs. Figure 9d [157] further visualizes correlated Li^+^ motion and lattice distortion in LiFePO_4_: time-resolved atomic-resolution scanning transmission electron microscopy (STEM) images of a Li^+^ diffusion channel along [010] reveal transient changes in Li site occupancy, Fe^2+^ → Fe^3+^ oxidation and polaron formation, and locally narrowed diffusion pathways that reduce Li^+^ diffusivity, providing a direct real-space picture of ion transport-structure coupling in olivine phosphates.

In summary, advanced characterization techniques are redefining the understanding of ion transport and degradation in LROs by offering multiscale, time-resolved insights into electrochemical and structural evolution. As summarized in Table 3, the synergistic application of CV, EIS, GITT, operando ND, and in situ TEM enables comprehensive analysis of kinetic, thermodynamic, and structural factors governing battery performance, and directly links local cation rearrangements and phase transitions to macroscopic voltage fade and capacity retention. As data complexity grows, the integration of machine learning and first-principles modeling will be critical for deciphering hidden correlations and accelerating material discovery. The convergence of real-time experimentation and intelligent data analysis heralds a transformative era for LIBs development, with significant implications for energy density, rate capability, and lifetime enhancement.

### Atomistic Simulation Technology

In addition to crystal-structure constraints that arise from diffusion path geometry and network connectivity, sluggish Li^+^ diffusion in LROs can be governed by a limited set of electronic-structure-related factors that reshape the migration energy landscape. First-principles calculations quantify these factors using physically meaningful descriptors and provide a basis for narrowing kinetic design from non-specific modification strategies toward targeted tuning. In practice, DFT combined with the nudged elastic band (NEB) method is widely used to map minimum-energy pathways and activation barriers, which is essential for LROs because the same hop can exhibit distinct barriers in different local 0-TM to 3-TM environments and in the presence of defects or interfacial fields.

A first mechanistic factor is the electrostatic repulsion associated with the tetrahedral activated configuration during the o-t-o hop in the Li layer. Along this pathway, the tetrahedral intermediate shares faces with adjacent TM octahedra, and the Li^+^-migration barrier increases with the effective cationic charge and the local electrostatic potential around the tetrahedral site. This effect can be quantified using Bader charges of face-sharing cations, site-projected electrostatic potentials, and NEB-resolved activation barriers across local 0-TM, 1-TM, 2-TM, and 3-TM environments [39, 159]. These descriptors motivate targeted strategies that reduce short-range electrostatic repulsion near the activated configuration, including controlled cation ordering that maximizes 0-TM percolation, judicious low-valence or more covalent dopants placed at face-sharing positions, and lattice-expansion approaches that increase Li slab spacing while avoiding irreversible reconstruction.

A second mechanistic factor is redox-induced charge redistribution coupled with Li motion, which becomes pronounced under deep delithiation and during anionic redox. Localized hole states on oxygen or transition metals can drive local lattice relaxation and modify the potential energy surface for Li migration. DFT-accessible descriptors include the O 2*p* band center relative to the Fermi level, quantitative TM-O covalency obtained from projected density of states or bond analyses, and the degree of hole localization characterized by charge-density metrics and polaron formation energies. For example, regulating oxygen covalent electron localization has been proposed as a design concept to improve anionic redox reversibility in LROs, offering a practical route to mitigate excessive charge localization that can deteriorate kinetics [160]. In addition, first-principles analyses in layered oxides have shown that small-polaron formation and charge localization can correlate with Li migration behavior, underscoring the necessity of electronic descriptors when interpreting ionic transport beyond purely geometric considerations [161]. These insights motivate kinetic tuning through electronic-structure modulation, such as adjusting covalency to avoid overly localized hole states while retaining reversible oxygen redox.

A third mechanistic factor is defect-modulated local electrostatics and bonding, including Li/TM antisites, oxygen vacancies, stacking faults, and the onset of O–O dimerization. Such defects introduce spatial variations in local potential and short-range bonding, which can create trapping sites or high-barrier constrictions for Li hopping, and they can also nucleate oxygen dimer formation in highly delithiated states. First-principles descriptors include defect formation energies and charge transition levels, defect association (binding) energies with Li vacancies, local migration barriers in the vicinity of defects, and statistical distributions of O–O distances as indicators of dimerization propensity [162, 163]. For example, an oxygen vacancy located near the Li layer has been reported to reduce the Li migration barrier from 0.69 to 0.39 eV in DFT calculations, illustrating that controlled defect chemistry can lower kinetic barriers when the relevant defect configurations are thermodynamically accessible [164, 165]. Meanwhile, defect-driven oxygen dimerization has been discussed from a first-principles perspective in layered oxide frameworks, providing descriptor-based criteria to evaluate whether an attempted kinetic enhancement may increase the risk of irreversible anion chemistry [162, 163]. Targeted solutions therefore include regulating defect populations via oxygen chemical potential and dopant selection, as well as engineering defect gradients that reduce Li^+^ barriers while preserving lattice oxygen stability [162–165].

A fourth mechanistic factor arises at interfaces, where electronic alignment can form space-charge regions that redistribute Li vacancies and alter the effective migration barrier near surfaces and coating layers. DFT descriptors such as interfacial band alignment, work-function mismatch, interfacial dipoles, and the Li chemical-potential profile across the interface provide a direct basis for screening coatings and heterostructures. For example, computation-guided screening has been used to identify coating materials and dopant chemistries that reduce interfacial transport resistance while maintaining chemical compatibility, enabling a descriptor-based route for interface selection rather than relying on generic coating choices [166]. In this view, preferred coatings are those that provide low Li transport barriers and also produce a favorable electrochemical potential landscape that mitigates interfacial Li depletion while stabilizing lattice oxygen [166].

**Fig. 10:**
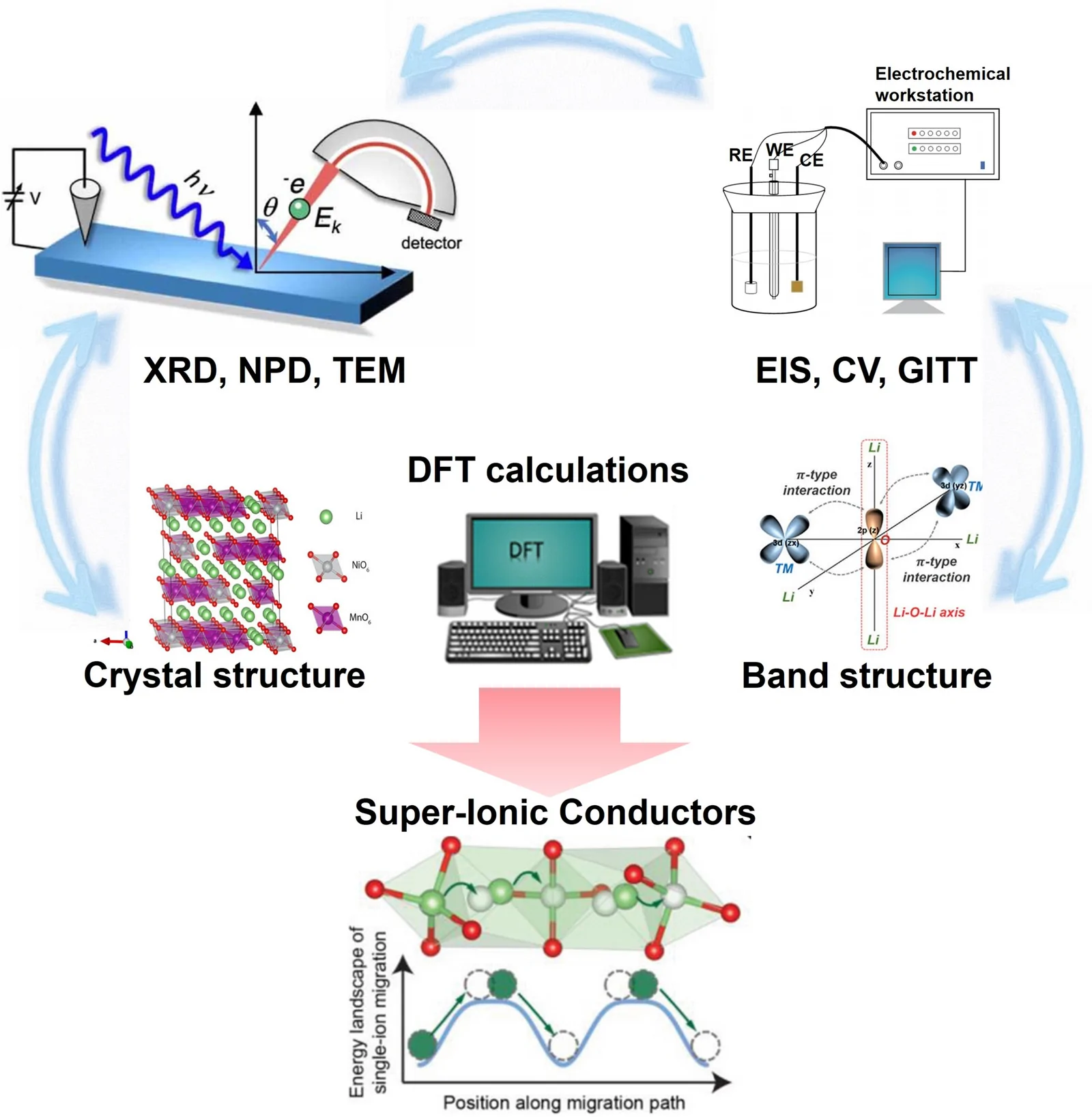
DFT-driven optimization of ultra-high Li^+^ conductive materials [173–175]

To accelerate exploration beyond what is feasible by direct NEB sampling alone, high-throughput density functional theory (HT-DFT) enables rapid evaluation of kinetic descriptors over broad compositional and structural spaces, and these outputs can be coupled with machine learning (ML) models for scalable screening [167, 168]. An illustrative example is summarized in Fig. 10, which presents a DFT-driven optimization workflow for identifying ultra-high Li^+^ conductive materials and narrowing candidate spaces using first-principles descriptors and screening criteria. To maintain interpretability and avoid generic correlations, a practical strategy is to use the physics-based descriptor sets associated with the mechanistic factors above as model inputs, including transition-state electrostatic indicators, covalency and band descriptors, and defect formation energies, rather than relying on purely empirical compositional features. Graph neural networks (GNNs) are well suited for representing local environments in complex cathode lattices and have been increasingly adopted for materials-property prediction [169–171]. For kinetic problems, mechanistically informed node and edge features constructed from first-principles descriptors can improve both data efficiency and physical plausibility. Model interpretability methods such as Shapley additive explanations (SHAP) can then be used to identify which descriptors and local motifs most strongly control predicted diffusion metrics, providing feedback for targeted synthesis and structural design [172].

While data-driven approaches have advanced rapidly for predicting Li^+^ transport, several challenges remain. The reliability of ML and GNN models is strongly influenced by the quality, diversity, and physical fidelity of the training data, which can be constrained by DFT-level approximations and the limited availability of experimentally validated diffusion datasets. In addition, model transferability across different chemistries, defect populations, and operating conditions remains non-trivial for LROs, where lattice, electronic, and interfacial effects are strongly coupled. Accordingly, current practice increasingly emphasizes the use of physics-informed descriptor sets, uncertainty quantification, and interpretability analysis to maintain physical consistency beyond purely correlative prediction. Importantly, data-driven models are most effective when embedded in a closed-loop workflow that integrates computation with experimental validation and operando characterization, allowing iterative refinement of descriptors, datasets, and design rules toward high-performance, fast-charging LIB cathodes.

## Summary and Outlook

### Multiscale Strategies and Challenges

Despite considerable advances in the structural design and compositional tuning of LROs, a fundamental contradiction remains unresolved: The redox processes responsible for high capacity simultaneously destabilize the structural framework and affect Li^+^ transport kinetics. The strong coupling between these processes and lattice evolution progressively increases Li^+^ migration barriers, thereby converting beneficial charge-storage reactions into degradative pathways. These instabilities are not confined to the atomic scale but extends to mesoscale and electrode levels, where particle fracture, interfacial degradation, and chemo-mechanical feedback loops further accelerate capacity loss. Consequently, Li^+^ diffusivity in LROs should be regarded not as an intrinsic property but as a dynamic parameter governed by coupled structural and chemical evolution across multiple length scales.

To address these challenges, various multiscale engineering strategies have been explored. At the atomic scale, lattice and redox-state engineering achieved through targeted doping or cation disorder can stabilize low-barrier diffusion channel and suppress irreversible structural transformations. At the particle level, morphology optimization mitigates intergranular stress and promotes homogeneous transport. At the interface, nanoscale coatings and defect engineering improve electrochemical stability and regulate local ionic conductivity. However, these methods are often applied in isolation, providing partial benefits but lacking a unified strategy to coordinate degradation processes across scales.

### Key Principles and Future Directions

Building on these advancements, overcoming intrinsic transport limitations requires a shift toward a mechanism-driven paradigm. Rather than treating Li^+^ mobility as a passive outcome of redox chemistry, ionic transport should be actively modulated by reconstructing the migration energy landscape. This involves designing redox-reversible lattice frameworks, stabilizing low-barrier motifs, and eliminating kinetic bottlenecks, enabling coordinated control of both transport and structural evolution. Achieving this paradigm requires deep integration across theory, computation, and characterization. Multiscale modeling can bridge quantum-level descriptors with mesoscale chemo-mechanical behavior, providing predictive insights into transport limitations. Rational materials design, including high-entropy oxides, metastable lattices, and defect-engineered frameworks, offers pathways to enhance both ionic conductivity and structural robustness. Simultaneously, standardized operando techniques integrated with time- and spatially-resolved probes provide causal mapping between structure evolution and transport properties.

To make these advances practical, specific performance targets for fast-kinetic LROs in real-world cells need to be established. These targets include: an areal capacity of 5–10 mAh cm^−2^, balancing high energy density with practical device size; C-rates of 2 –5 C to support fast charging, aiming for 80% capacity in 30 min without compromising structural stability; and cycle life targets of 1000–1500 cycles, with less than 20% capacity degradation, ensuring long-term performance under rapid charge/discharge conditions. Achieving these goals requires optimizing both the intrinsic material properties and the structural motifs that govern ionic transport and stability. These performance targets are closely linked to the multiscale design and modeling strategies. By leveraging DFT/ML/GNN-guided optimization, structural motifs can be systematically screened and refined to meet these practical requirements, creating a direct link between fundamental design principles and rate performance. This approach will enable the development of fast-kinetic LROs that deliver both high energy density and fast-charging capability, while maintaining structural integrity over extended cycling.
